# Forecasting accuracy is not enough: safety-oriented evaluation of neural digital twins prior to *in-silico* insulin and carbohydrate intervention in type 1 diabetes

**DOI:** 10.3389/fendo.2026.1958918

**Published:** 2026-10-14

**Authors:** Ahmed Ibrahim, Ameen Banjar, Simon Harper, Ala Alarood, Eesa Alsolami

**Affiliations:** 1 Department of Computer Science and Artificial Intelligence, College of Computer Science and Engineering, University of Jeddah, Jeddah, Saudi Arabia; 2 Department of Information Systems and Technology, College of Computer Science and Engineering, University of Jeddah, Jeddah, Saudi Arabia; 3 Department of Computer Science, The University of Manchester, Manchester, United Kingdom; 4 Department of Information Technology, College of Computer Science and Engineering, University of Jeddah, Jeddah, Saudi Arabia; 5 Department of Cybersecurity, College of Computer Science and Engineering, University of Jeddah, Jeddah, Saudi Arabia

**Keywords:** continuous glucose monitoring, digital twin, dose-response audit, glucose forecasting, *in-silico* intervention simulation, LSTM, treatment-response plausibility, type 1 diabetes

## Abstract

Neural networks trained on a single patient’s continuous glucose monitoring (CGM) record are increasingly proposed as “digital twins” that could let clinicians rehearse insulin and carbohydrate decisions in silico before applying them to the patient. Before any such use, a candidate twin should respond to treatment in a direction consistent with established pharmacology: a model that merely extrapolates the recent glucose trend can report an excellent forecasting error while remaining effectively blind to insulin, and this failure is invisible to the root-mean-square error (RMSE) that dominates the glucose-forecasting literature. We examined this gap in two independent cohorts: a longitudinal, multimodal University of Manchester cohort (17 participants enrolled; 13 formed the Core analytical cohort after data-quality screening) and, as an independent replication of the protocol, the OhioT1DM dataset (12 participants). Patient-specific two-layer long short-term memory (LSTM) models were trained under a strict chronological 60/20/20 split and evaluated across five random seeds. A physiology-guided variant added insulin-on-board and carbohydrate-on-board states with a double-monotonicity training penalty encouraging insulin to lower and carbohydrate to raise predicted glucose. Two findings replicated across both cohorts and all seeds. First, on the accuracy axis, a physiology-free Vanilla LSTM had the lowest one-step RMSE (Manchester 7.00, Ohio 5.02 mg/dL; Friedman *χ*^2^(5) = 43.4 and 46.7, both *p <* 10*^−^*^7^), and under two-hour rollout a non-learning persistence baseline beat every neural model at 60 and 120 minutes—even after rollout-aware training—so ranking twins by accuracy alone would select a model that cannot represent treatment at all. Second, on a graded insulin dose-response audit, the physiology-guided model produced the steepest correctly-directed (glucose-lowering) response among the primary configurations in both cohorts (slope significantly better than the unconstrained model: Holm-corrected *p* = 0.002 and 0.003), whereas removing the directional constraint left a wrong-signed response; a supplementary ablation without masking was steeper still, pointing to the directional penalty, not masking, as the driver. Its advantage over the simpler Vanilla model was significant on for this cohort dependence. Accuracy and treatment-response plausibility are separable properties; we provide a reproducible protocol auditing both before a neural twin is used for intervention simulation.

## 1 Introduction

Type 1 Diabetes (T1D) management is shaped by a continuous balance between hyperglycaemia and hypoglycaemia risk. Clinical standards emphasise individualised glycaemic targets, CGM-informed care, technology-enabled monitoring, and explicit reporting of clinically meaningful low-glucose thresholds (1, 2). Neural digital-twin candidates are therefore attractive because they offer the possibility of testing glucose trajectories and treatment perturbations in silico before any real-world intervention is considered. However, the term “digital twin” implies physiological fidelity that must be verified beyond observational forecasting accuracy; a model that tracks glucose well may still fail to respond in the physiologically expected direction when insulin or carbohydrate inputs are deliberately changed.

The central methodological problem is that accurate glucose forecasting is not equivalent to a physiologically credible digital twin. CGM time series are highly autocorrelated, especially at short horizons, which is precisely why simple time-series predictors are strong short-horizon baselines for glucose (49). A recurrent neural network can therefore minimise RMSE by copying recent glucose history while under-learning the delayed effects of insulin, carbohydrate absorption, and behavioural context. This is an instance of shortcut learning: a model exploits a predictive association that works on the validation distribution but fails when the evaluation setting changes (3).

We refer to this failure mode as *autoregressive physiological insensitivity*: a model behaviour in which recent CGM history dominates the learned representation, producing low observational forecasting error while leaving the model insufficiently responsive to therapeutically meaningful sulin and carbohydrate perturbations. This term is used descriptively rather than diagnostically; it denotes a model-evaluation failure mode, not a clinical property of any participant.

This distinction matters in T1D because observational associations can invert therapeutic meaning. Insulin boluses often occur when glucose is already high or rising. A purely data-driven model may then learn that bolus insulin is associated with future hyperglycaemia, rather than learning that insulin should, after pharmacodynamic delay, exert glucose-lowering pressure. Recent glucose-forecasting and digital-twin research has begun to address this by incorporating mechanistic structure, knowledge guidance, and scenario-simulation reasoning (4–6).

A second issue is cohort fairness. Multimodal T1D datasets rarely contain complete streams for every participant. A model evaluated on glucose-insulin-nutrition data may use a different participant set from a model evaluated on sleep, wearable, and contextual data. Without explicit cohort stratification, the model comparison is confounded by participant availability, missingness, and selection. Clinical machine-learning failures often arise not only from model architecture but also from opaque data generation, eligibility criteria, and deployment mismatch (7, 8).

Despite the rapid growth of digital-twin proposals for T1D, these models are still validated almost exclusively with forecasting-error metrics. To our knowledge, prior studies have not jointly evaluated forecasting accuracy, matched-cohort comparison, and patient-level graded perturbation responsiveness under a single held-out protocol; that combination is the gap we address. Our central empirical finding is that these two properties come apart sharply on real patient data: a trivial non-learning baseline that simply holds the last glucose value has lower RMSE than every neural configuration we trained at the 60- and 120-minute horizons, yet that same baseline is by construction incapable of representing any treatment effect at all. A model can thus perform well on the metric the field currently trusts while remaining unsuitable for the in-silico intervention testing that motivates digital twins in the first place.

Building on this finding, the paper makes five contributions aimed at safer pre-deployment screening. First, it introduces and formally defines *cohort-fair* comparison, evaluating competing model configurations on the identical set of patients so that apparent differences cannot be an artefact of which patients happened to carry each data stream—a confound we show can otherwise reverse conclusions. Second, it operationalises *directional treatment-response plausibility* as a directly measurable quantity through a graded insulin and carbohydrate dose-response audit, separating it from forecasting accuracy as an independent evaluation axis rather than assuming accuracy implies plausibility. We use “plausibility” throughout in this specific, deliberately modest sense—whether a candidate’s predicted response moves in the pharmacologically correct *direction* under a perturbation—and not to claim that the simulated response’s magnitude, timing, or kinetics have been validated as physiologically realistic; the audit is best understood as a preliminary behavioural screening step, not a safety validation. Third, it uses a structured ablation to identify which physiological ingredients (glucose masking, pharmacodynamic on-board states, and directional monotonicity penalties) actually shift this accuracy–plausibility balance, providing concrete guidance for building twins that are screened rather than merely fitted. Fourth, it establishes the robustness of these findings through independent replication of the entire protocol on a second cohort (OhioT1DM) and across five random seeds, and controls for the one-step-training/multi-step-rollout mismatch with a rollout-aware training comparison. Fifth, it releases a reproducibility package through GitHub and Zenodo so that the evaluation protocol, figures, and pipeline can be independently rerun and reused as a screening template for other candidate twins (9–11).

## 2 Related work

### 2.1 Glucose forecasting in type 1 diabetes

Data-driven glucose forecasting has progressed from classical autoregressive methods to recurrent, multi-scale, and attention-based neural architectures. A recent survey covering 2015–2023 shows that LSTM-type approaches are common because they can represent nonlinear temporal dependencies in CGM data, and further categorises forecasting approaches by input modality (CGM, insulin, meal, and activity streams) and by modelling paradigm (physiological, data-driven, and hybrid) (22), providing a broad contemporary backdrop against which the present cohort-fair, multi-stream evaluation is positioned. Representative models include multi-scale LSTM networks, stacked recurrent architectures with smoothing, and therapy-driven forecasting designs that explicitly use therapeutic inputs rather than CGM history alone (12–14). Feature-ablation work further shows that input selection can substantially affect glucose forecasting outcomes (15).

A separate and more recent line of work moves beyond patient-specific recurrent networks entirely, towards direct multi-horizon, population-pretrained, and attention-based architectures. TimeGPT, a general-purpose time-series foundation model, has been applied zero-shot (without patient-specific fine-tuning) to paediatric T1D glucose forecasting (24). Transformer architectures have been proposed both for direct glucose-level and hypo-/hyperglycaemia-event forecasting (25) and, using a temporal fusion transformer trained on the same OhioT1DM dataset used here, for population-specific prediction with an edge-deployment target (26). A related transformer-based approach, GPFormer, targets direct multi-horizon prediction with meta-learning for zero-shot domain generalisation across populations, evaluated in part on OhioT1DM alongside other clinical cohorts (27). At the representation-learning end of this spectrum, CGMformer is pretrained with masked self-supervised learning on a large multi-participant CGM corpus to produce a general-purpose glucose-dynamics representation, subsequently applied to screening and dietary-response tasks rather than direct multi-step forecasting (28). These approaches differ from the present study along every axis relevant to the RMSE-paradox finding below: they are (i) population-pretrained or zero-shot rather than patient-specific, (ii) architecturally direct-multi-horizon or attention-based rather than a one-step-trained recurrent network evaluated under autoregressive rollout, and (iii) not evaluated here against a treatment-response plausibility audit. Because the RMSE paradox reported in this paper is a consequence of autoregressive error accumulation under one-step training, it need not generalise to architectures that predict multiple horizons directly or that never re-feed their own predictions; we therefore do not claim, and this paper cannot test, whether non-learning persistence would similarly outperform these modern approaches.

Despite these advances, RMSE-focused evaluation is insufficient when the model is intended to support in-silico intervention analysis. A model can be accurate on observed data yet fail to respond in the physiologically expected direction to insulin or carbohydrate perturbations. This motivates the present paper’s use of both observational and interventional-style evaluations.

A patient-specific shallow neural network study by Kushner et al. (23) is methodologically close to the present work in two respects. First, their network architecture separates insulin-related inputs from non-insulin inputs in the first layer and constrains the insulin-related weights to act in a glucose-lowering direction, which is an architectural analogue of the directional constraint that the present paper enforces through the *L*_ins_ loss term rather than through weight-sign restrictions. Second, their train/test split enforces a four-hour gap around evaluation windows to reduce autocorrelation leakage between adjacent CGM samples, a concern directly analogous to the leakage-free training protocol adopted here. Their results also illustrate, for a 24-participant CSII/MDI cohort, the same qualitative risk addressed by the present paper: a model can achieve competitive RMSE (28–43 mg/dL across 60–240 minute horizons) while still being vulnerable to misattributing glucose rises to recent insulin dosing rather than to unmeasured meals. The present paper differs by treating this risk as a directly measurable evaluation axis (scenario simulation responsiveness) rather than addressing it solely through architectural design.

### 2.2 Mechanistic simulation, hybrid modelling, and digital twins

Mechanistic simulators have long provided a foundation for T1D in-silico testing. The Hovorka model and the UVA/Padova simulator encode physiologically interpretable glucose-insulin dynamics and have been used in closed-loop insulin-delivery research and preclinical simulation (16–18). More recent work has extended this tradition through digital twins, simulation-based inference, physiologically constrained neural frameworks, and real-world-data bolus-calculator development (6, 19, 20). The clinical relevance of digital-twin technology has also been demonstrated directly: a recent randomised trial mapped automated-insulin-delivery users to individual digital twins for biweekly therapy-parameter optimisation and what-if scenario replay, improving time-in-range and glycated haemoglobin over a six-month study (29). This trial illustrates that scenario-based digital-twin reasoning, of the kind evaluated diagnostically in the present paper, is already moving towards clinical practice, which strengthens the motivation for rigorously evaluating the physiological plausibility of candidate twins before any such use.

The present framework sits between mechanistic and data-driven traditions. It does not attempt to replace validated physiological simulators. Instead, it uses physiologically motivated constraints and pharmacodynamic latent states to test whether patient-specific neural twins respond in plausible directions under structured perturbations.

### 2.3 Multimodal context, missingness, and fairness

T1D glucose dynamics are affected by meals, insulin, sleep, activity, and physiological context. Sleep and activity are particularly relevant because they can shift insulin sensitivity and glycaemic variability (21). Mechanistic simulation work has shown that physical activity alters both insulin-independent glucose clearance and insulin sensitivity through distinct rapid and slow-decaying dynamics (30), underscoring that activity is not a negligible confound but a physiologically structured input that an evaluation framework should, in principle, account for. However, these streams are unevenly observed in free-living studies. Adding modalities can reduce the participant and change the population being evaluated. The Core-versus-Enriched design therefore treats cohort construction as an explicit methodological object rather than a background preprocessing detail.

## 3 Materials and methods

### 3.1 Data source and secondary-analysis scope

The primary analysis uses the University of Manchester longitudinal multimodal T1D dataset described by Alsuhaymi et al. (31). The source study reports multimodal monitoring of adults with T1D across CGM, insulin, nutrition, activity, and sleep-related streams. The present work is a secondary modelling analysis. It does not provide clinical treatment recommendations, does not claim prospective clinical validation, and should be interpreted as a methodological evaluation of digital-twin behaviour.

To test whether the findings generalise beyond a single cohort, every core analysis is repeated on an independent dataset, OhioT1DM (39, 44), which contains eight weeks of CGM, basal and bolus insulin, self-reported meals, and physiological band data for 12 adults with T1D (six released in 2018 and six in 2020). OhioT1DM was chosen because it is the closest public analogue to the Manchester cohort in multimodal richness while originating from a different country, clinical setting, and device generation, making it a strong independent-cohort replication of the protocol rather than a re-sampling of the same population. The two cohorts are processed and evaluated under one identical protocol (Section 3.6); the only intentional differences are documented in Section 3.11. To be explicit about the asymmetric role the two cohorts play: Manchester is the *development cohort*, on which the architecture, features, and regularisation settings were designed and frozen (Section 3.6); OhioT1DM is a *held-back confirmation cohort*, touched only after that protocol was frozen and used solely to test whether the frozen protocol’s findings reproduce under a new, unseen population. Throughout the paper, phrases such as “replicated across both cohorts” describe this development/confirmation relationship and should not be read as claiming the two cohorts carry equal evidential weight or an independently pre-registered symmetric design.

### 3.2 Cohort construction

Participants were stratified before evaluation. The Core cohort required usable glucose, basal insulin, bolus insulin, and nutrition streams. The Enriched cohort required all Core streams plus sleep timeline and sleep-summary data. This produced *N* = 13 Core participants, *N* = 10 Enriched participants, and an overlapping *N* = 10 subgroup for paired Core-versus-Enriched comparison. **Table 1** reports participant-level modality availability and cohort assignment.

**Table 1:** Participant-level modality availability and cohort assignment.

PID	Core streams	Sleep streams	Core	Enriched	Reason or note
2301	Yes	Yes	Included	Included	Full data
2302	Yes	Yes	Included	Included	Basal long-acting events retained
2303	No	Yes	Excluded	Excluded	Missing basal, bolus, and nutrition streams
2304	Yes	Yes	Included	Included	Full data
2305	Yes	No	Included	Excluded	Missing sleep timeline
2306	Yes	Yes	Included	Included	Basal long-acting events retained
2307	Yes	Yes	Included	Included	Full data
2308	Yes	Yes	Included	Included	Full data
2309	Yes	Yes	Included	Included	Full data
2310	No	No	Excluded	Excluded	Missing nutrition stream
2313	Yes	Yes	Included	Included	Full data
2314	Yes	No	Included	Excluded	Missing sleep timeline
2320	No	No	Excluded	Excluded	Basal-only or bolus-only data insufficient
2401	Yes	Yes	Included	Included	Full data
2403	Yes	No	Included	Excluded	Missing sleep timeline
2404	No	No	Excluded	Excluded	Basal-only or bolus-only data insufficient
2405	Yes	Yes	Included	Included	Full data

We define a *cohort-fair comparison* as a model comparison in which competing feature configurations are evaluated on the same participant subset whenever modality availability differs, so thatperformance differences are not confounded by participant eligibility. Without this constraint, a model trained on glucose-insulin-nutrition data and a model trained on sleep and wearable context might be compared on different subsets, making it impossible to attribute performance differences to the features rather than to the participants. All Core-versus-Enriched comparisons in this paper use the overlapping *N* = 10 subgroup to satisfy this definition.

### 3.3 Participant demographics and glycaemic characteristics

**Table 2** reports body composition, therapy parameters, and monitoring duration for the 13 Core participants. HbA1c values were not available in the source dataset. **Table 3** reports participant-level time-in-range metrics derived from the CGM records.

**Table 2:** Participant demographics and therapy parameters for the Core cohort (*N* = 13).

PID	Weight (kg)	BMI	Therapy	ICR (g/U)	ISF (mg/dL/U)	Days	Enriched
2301	64	24.4	CSII	9.7	72.7	109	Yes
2302	61	20.1	MDI	7.3	25.4	169	Yes
2304	82	30.1	CSII	3.3	25.0	116	Yes
2305	60	22.9	MDI	9.3	31.5	63	No
2306	63	23.4	MDI	6.0	31.5	102	Yes
2307	62	21.5	CSII	15.0	60.1	29	Yes
2308	70	22.3	CSII	11.1	40.3	106	Yes
2309	103	36.5	CSII	13.7	28.6	85	Yes
2313	127	38.3	MDI	5.6	14.0	99	Yes
2314	86	25.1	MDI	9.7	24.1	91	No
2401	85	25.7	MDI	3.8	14.6	91	Yes
2403	70	22.9	MDI	15.3	24.9	116	No
2405	87	25.4	MDI	9.0	24.9	98	Yes
Mean	78.5	26.0	5 CSII/8 MDI	9.1	32.1	98	
Range	60–127	20.1–38.3		3.3–15.3	14.0–72.7	29–169	

ICR, insulin-to-carbohydrate ratio (g/U); ISF, insulin sensitivity factor (mg/dL per unit). CSII, continuous subcutaneous insulin infusion (pump); MDI, multiple daily injections. HbA1c was not available in the source dataset. Values are as recorded in the study parameter file.

**Table 3:** CGM-derived glycaemic metrics for the Core cohort (*N* = 13), following the international consensus thresholds on time in range (43).

PID	CGM readings	Mean glucose (mg/dL)	TIR (%)	TAR (%)	TBR (%)
2301	31,680	149.1	77.5	21.8	0.7
2302	48,809	133.7	86.7	10.9	2.4
2304	33,529	143.4	80.3	19.1	0.6
2305	18,431	183.7	48.7	48.0	3.3
2306	29,527	128.5	82.3	13.0	4.7
2307	8,535	166.8	66.7	32.3	1.0
2308	30,623	126.0	84.0	12.3	3.6
2309	24,651	182.6	47.8	50.8	1.4
2313	28,676	177.9	51.1	42.7	6.2
2314	26,320	167.0	62.8	36.8	0.4
2401	26,492	135.8	71.5	19.1	9.4
2403	33,567	162.5	60.7	35.9	3.5
2405	28,419	157.1	64.1	33.1	2.8
Mean	28,404	157.3	68.0	28.9	3.1

TIR, time-in-range 70–180 mg/dL; TAR, time above range >180 mg/dL; TBR, time below range <70 mg/dL. All values are percentages of valid CGM readings. Mean glucose is in mg/dL. Metrics are computed from the full valid raw CGM record before model scaling and are reported descriptively to characterise observed glycaemia during the monitoring period.

Glycaemic control varied substantially across participants. Mean TIR ranged from 47.8% (participant 2309) to 86.7% (participant 2302), with a cohort mean of 68.0%. Participant 2313 had the highest BMI (38.3 kg/m^2^), the highest weight (127 kg), and among the lowest TIR (51.1%); this single-participant co-occurrence is reported descriptively and is not evidence of a causal or general relationship between metabolic load and glycaemic variability in this cohort. Participant 2307 had the shortest monitoring record (29 days), which is noted as a source of potential instability in patient-specific model training. Therapy types were distributed as 5 CSII (pump) and 8 MDI across the Core cohort.

### 3.4 Data preprocessing, temporal alignment, and quality control

Both cohorts were mapped onto a common processed schema so that one modelling and evaluation pipeline could run unchanged on each. Every stream was resampled to a regular 5-minute grid spanning each participant’s record; duplicate timestamps were removed, keeping the first occurrence; and no CGM value was extrapolated beyond the observed record. For OhioT1DM, CGM values were snapped to the nearest grid point within a 2-minute tolerance; interior gaps of up to 30 minutes (six steps) were linearly interpolated, and longer gaps were left missing and the affected grid rows were dropped, so no OhioT1DM window was built across a sustained sensor outage. Pooled across all Ohio participants, 1.59% of retained grid rows were interpolated rather than observed (participant range 0.46–2.79%; **Supplementary Table S9a**); re-evaluating the existing rollout RMSE checkpoints after excluding every window whose target lands on an interpolated point leaves results materially unchanged at every horizon and configuration (largest shift 0.33 mg/dL at 120 minutes; **Supplementary Table S9b**), so the paper’s long-horizon conclusion is robust to excluding interpolation-affected targets. For Manchester, missing or zero-valued readings on the common 5-minute grid were carried forward, then backward, from the nearest valid neighbouring reading; because a minority of Manchester participants had a native CGM sampling rate coarser than 5 minutes, this fill step is used more heavily for them, which is one reason OhioT1DM—where interpolation is capped and independently confirmed not to alter the persistence-related finding above—serves as the confirmatory cohort for that result.

Discrete events were binned into their 5-minute interval: bolus doses and meal carbohydrates were summed within each bin (so co-occurring events add), and each participant’s programmed basal rate was expanded as a step function held forward until the next programmed change, with temporary-basal periods overriding the programmed rate for their stated interval. For the OhioT1DM MDI-equivalent handling, long-acting basal events were represented through the same basal-rate channel rather than as bolus impulses. Pharmacodynamic on-board states were then derived from these aligned event series by the exponential recursions in Equations 3, 4 (*τ*_ins_ = 180 min, *τ*_carbs_ = 120 min); we describe these as pharmacodynamic *exposure proxies* rather than full pharmacokinetic models, since they capture accumulated, decaying exposure but not absorption lag, peak timing, insulin-type differences, or meal-composition effects. Circadian phase was encoded as sine/cosine of time-of-day and a weekend indicator was added. The Core feature set is the eight resulting channels (glucose, insulin-on-board, basal rate, meal carbohydrates, carbohydrate-on-board, two circadian terms, weekend); the Manchester Enriched set appends three contextual channels (heart rate, step count, sleep efficiency), each resampled to the same grid and forward-filled between observed readings, with sleep efficiency held piecewise-constant across each scored sleep interval; we did not impose an explicit maximum fill duration, so any residual staleness in low-sampling-rate periods is a limitation of the Enriched channels rather than the Core feature set used for the headline results.

**Supplementary Table S1** reports, for every participant, the recording span, the number of usable 5-minute samples, the counts of bolus and meal events, and the number of valid input windows in each of the training, validation, and test segments (a window requires 72 consecutive present rows plus its prediction target). This makes explicit that the Core-versus-Enriched contrast in Section 4 is computed on the same participants and comparable window counts, so it reflects the added information rather than differential missingness.

### 3.5 Statistical analysis

This section states the pre-specified analysis plan; all numerical results appear in Section 4.Tests were implemented in Python with SciPy (37), and the analysis was run identically on both cohorts.

#### 3.5.1 Unit of inference

The unit of analysis is the participant. Each participant contributes one value per configuration for every endpoint, obtained by averaging over that participant’s evaluation windows; the multiple windows and the five random seeds are treated as within-participant technical replication, not as independent samples. Seed variation is reported as thespread across seeds and as the number of seeds (of five) preserving each ordering, never as an inflated sample size.

#### 3.5.2 Planned tests

For each inferential endpoint (one-step RMSE; multi-horizon rollout RMSE;the insulin dose-response slope; and the carbohydrate dose-response violation count) the model configurations are compared with a Friedman omnibus test across participants as repeated-measures blocks, with Kendall’s *W* as the omnibus effect size. When the omnibus test is significant, the three pre-specified pairwise contrasts (Full Core vs. Vanilla, Full Core vs. NoMono, Vanilla vs. NoMono) are tested with two-tailed Wilcoxon signed-rank tests, with matched-pairs rank-biserial correlation as the pairwise effect size (38) and Holm correction applied across the three comparisons. The paired Core-versus-Enriched contrast uses a Wilcoxon signed-rank test on the overlapping subgroup with the same effect-size definition.

#### 3.5.3 Multiplicity and primary horizon

For the multi-horizon rollout analysis the 120-minute horizon is the pre-specified primary endpoint; 5, 30, and 60 minutes are secondary, and the four per-horizon omnibus tests are Holm-corrected. For insulin treatment-response plausibility, the pre-specified inferential endpoint is the participant-level dose-response slope; sign-correctness and monotonicity are secondary, descriptive components reported alongside it in **Tables 4**, **5** but not subjected to separate confirmatory tests. The carbohydrate audit uses the participant-level monotonicity-violation count (out of 3 dose transitions) as a secondary inferential endpoint, analysed with the same Friedman procedure across the three primary configurations (Section 4.3).

**Table 4:** Insulin dose-response plausibility by configuration, participant-level mean (*± *SD) across five seeds.

Cohort	Manchester (*N* = 13)			OhioT1DM (*N* = 12)		
Configuration	Slope	Sign	Mono	Slope	Sign	Mono
Full Core	-5.24* ± *3.63	0.88	0.92	-3.37* ± *1.50	0.93	0.95
VanillaLSTM	-1.98* ± *4.48	0.68	0.71	-0.52* ± *3.95	0.72	0.74
NoMono	0.07* ± *5.93	0.65	0.57	1.70* ± *5.40	0.56	0.50

Sign: fraction of doses lowering glucose. Mono: monotonicity score (1 = perfectly monotone). The slope is the primary plausibility endpoint. Slope: OLS slope of 2-hour glucose change on dose (mg/dL per U; negative = directionally plausible).

**Table 5:** Additional insulin dose-response plausibility for the remaining ablations (NoMask, NoIOB, NoCOB), participant-level mean (*± *SD) across five seeds, computed against the corrected checkpoints.

Cohort	Manchester (*N* = 13)			OhioT1DM (*N* = 12)		
Configuration	Slope	Sign	Mono	Slope	Sign	Mono
NoMask	*−*7.06* ± *3.75	0.84	0.95	*−*7.27* ± *2.27	0.91	1.00
NoCOB	*−*5.85* ± *3.24	0.81	0.94	*−*3.57* ± *2.06	0.88	0.88
NoIOB	*−*2.75* ± *2.00	0.79	0.87	*−*1.52* ± *1.74	0.70	0.70

Columns as in **Table 4**. NoIOB and NoCOB use configuration-specific perturbation logic to match their feature layout (NoIOB: the substituted Raw_Bolus channel is perturbed as a one-step impulse, not a decaying reservoir like IOB; NoCOB: 7 features, no COB_g channel).

#### 3.5.4 Uncertainty

Uncertainty is quantified by a hierarchical, two-level bootstrap that resamples participants with replacement and then, within each resampled participant, resamples their five per-seed values with replacement (10,000 replicates; 95% CIs from the 2.5th/97.5th percentiles), preserving the clustered participant-then-seed structure of the design, and by the dispersion of each endpoint across the five seeds more generally. Seeds are resampled *within*, never across, participants because the five seeds represent stochastic technical variability from a single participant’s own model (random weight initialisation and mini-batch ordering under an otherwise identical training protocol), not five additional independent participants; pooling seeds with participants would understate the true between-participant uncertainty by treating technical replication as if it were biological/behavioural variation. A significance threshold of α = 0.05 is used throughout.

### 3.6 Model architecture and training

#### 3.6.1 Input feature representation

Each observation at CGM time step *t* is represented as the feature vector shown in Equation 1

(1) $$x_{t}={\left[{ɡ}_{t},\;{\text{IOB}}_{t},\;r_{t}^{\text{bas}},\;C_{t},\;{\text{COB}}_{t},\text{ sin }(\omega_{\text{day}}t),\text{ cos }(\omega_{\text{day}}t),\;w_{t}\right]}^{⊤},$$

where ${ɡ}_{t}$ is CGM glucose concentration (mg/dL), ${\text{IOB}}_{t}$ is insulin-on-board, $r_{t}^{\text{bas}}$ is the basal insulin rate (U/hr), $C_{t}$ is meal carbohydrates (g), ${\text{COB}}_{t}$ is carbohydrates-on-board, $\omega_{\text{day}}t=2\pi t/288$ encodes the 24-hour circadian cycle at 5-minute resolution (288 steps/day), and $w_{t}\in \{0,1\}$ is a weekend indicator. The input dimension is d=8 for Core and d=11 for Enriched (which appends heart rate, step count, and sleep efficiency). A sliding window of L=72 consecutive steps (360 minutes) forms the model input, and the target is the glucose value h=1 step ahead in training and h∈{1,6,12,24} steps ahead in evaluation.

#### 3.6.2 Pharmacodynamic exposure-proxy computation

Insulin-on-board at time *t* is approximated by the exponential decay formulation in Equation 2, with rapid-acting insulin time constant $\tau_{\text{ins}}=180$ min:

(2) $${\text{IOB}}_{t}=\overset{t}{\underset{k=0}{\sum }}u_{k}\cdot e^{-(t-k)\text{Δ}t/\tau_{\text{ins}}},$$

where $u_{k}$ is the bolus dose delivered at step *k* and Δ*t* = 5 min is the CGM sampling interval. In practice this is computed by the efficient first-order recursion

(3) $${\text{IOB}}_{t}={\text{IOB}}_{t-1}\cdot e^{-\text{Δ}t/\tau_{\text{ins}}}+u_{t}.$$

Carbohydrates-on-board uses the same form with gastric-emptying time constant $\tau_{\text{carbs}}=120$ min (34):

(4) $${\text{COB}}_{t}={\text{COB}}_{t-1}\cdot e^{-\text{Δ}t/\tau_{\text{carbs}}}+m_{t},$$

where $m_{t}$ is the meal carbohydrate amount at step t. These states are pharmacodynamic *exposure proxies*: single-compartment exponential summaries of accumulated, decaying insulin and carbohydrate exposure that serve as informative inputs for theLSTM so that it can learn therapeutic effects rather than raw bolus-timing correlations (33). They deliberately do not model absorption lag, time-to-peak, insulin-type differences, multi-phase kinetics, or meal-composition effects, and should not be read as clinically exact insulin-on-board or carbohydrate-on-board quantities. For MDI participants, long-acting basal injections are represented through the basal-rate channel $r_{t}^{\text{bas}}$ as a sustained rate over the action window rather than as bolus impulses in Equation 3, so that rapid-acting IOB reflects only bolus insuli

#### 3.6.3 LSTM architecture

Each patient-specific model is a two-layer stacked LSTM (32) with hidden size 128 and inter-layer dropout rate 0.20:

(5) $$i_{t}=\sigma (W_{i}x_{t}+U_{i}h_{t-1}+b_{i}),$$

(6) $$f_{t}=\sigma (W_{f}x_{t}+U_{f}h_{t-1}+b_{f}),$$

(7) $${\tilde{c}}_{t}=\text{tanh}(W_{c}x_{\text{t}}+U_{c}h_{t-1}+b_{c}),$$

(8) $$c_{t}=f_{t}⊙c_{t-1}+i_{t}⊙{\tilde{c}}_{t},$$

(9) $$o_{t}=\sigma (W_{o}x_{t}+U_{o}h_{t-1}+b_{o}),$$

(10) $$h_{t}=o_{t}⊙\text{tanh }(c_{t}),$$

where σ(·) is the sigmoid function, ⊙ is element-wise multiplication, and $W_{\left\{i,f,c,o\right\}}$, $U_{\left\{i,f,c,o\right\}}$, $b_{\left\{i,f,c,o\right\}}$ are learnable weight matrices and bias vectors. The final hidden state $h_{T}$ of the last LSTM layer is projected through a single linear layer to produce the glucose forecast directly:

(11) $${\hat{ɡ}}_{t+h}=W_{\text{out}}h_{T}+b_{\text{out}}.$$

All downstream loss terms (Equations 13–15) are computed on ${\hat{ɡ}}_{t+h}$

#### 3.6.4 Stochastic glucose masking

During training only, the glucose channel of the input sequence is randomly zeroed to prevent the model from relying exclusively on recent CGM autocorrelation and encourage learning from the therapeutic inputs:

(12) $${\tilde{ɡ}}_{t}=\{\begin{array}{ll} {ɡ}_{t}\cdot 1[\xi_{t}>p_{\text{mask}}] & \text{with probability }p_{\text{apply}},\\ {ɡ}_{t} & \text{otherwise}, \end{array}$$

where *ξ_t_ ∼* Uniform [0,1] independently for each time step, $p_{\text{mask}}=0.60$ is the per-step masking probability, and $p_{\text{apply}}=0.40$ is the probability that the masking operation is applied to a given training batch. Masking is removed at inference time so that full CGM history is used for prediction.

#### 3.6.5 Double-monotonicity regularisation

Physics-guided training enforces two directional constraints simultaneously as a soft regularisation loss. Unlike architecturally monotone networks that guarantee monotonicity by construction through weight-sign or lattice constraints (45, 46), we impose direction through a soft training penalty, which is simpler to combine with the pharmacodynamic inputs and the masking scheme but—as our rollout analysis shows—transfers only partially to multi-step prediction. Given a mini-batch **X**, the model f(·) is evaluated on three versions of the input: the original, a version with IOB scaled up by factor 1.50, and a version with COB scaled up by factor 1.50. The insulin penalty enforces that more insulin-on-board should not increase the predicted glucose:

(13) $$L_{\text{ins}}=E[\text{ReLU}(f(X^{1.5\times \text{IOB}})-f(X))].$$

The carbohydrate penalty enforces that more carbohydrates-on-board should not decrease the predicted glucose:

(14) $$L_{\text{carbs}}=E[\text{ReLU}(f(X)-f(X^{1.5\times \text{COB}}))]$$

The total training loss combines the primary mean-squared-error objective with both monotonicity penalties:

(15) $$L_{\text{total}}=L_{\text{MSE}}+\lambda (L_{\text{ins}}+L_{\text{carbs}}),\;\lambda =0.10.$$

Both terms use ReLU(*·*) = max(0*,·*) so that only directional violations are penalised. The 1.5*×* scaling is defined in *raw physical units* of the IOB (or COB) channel, $x^{\prime }=1.5x$, not as a 1.5*×* multiplication of the standardised value z=(x−μ)/σ. Because the network consumes, standardised inputs applying the perturbation requires converting this raw-space scaling into the equivalent standardised-space update,

(16) $$z^{\prime }=1.5z+(1.5-1)\frac{\mu }{\sigma },$$

i.e. the standardised perturbation is the 1.5*×* scaling *plus* an additive offset proportional to the channel’s training-set mean and scale (*µ*, *σ* from the training-fitted StandardScaler), not *z^′^* = 1.5*z* alone. The additive offset is necessary because scaling only the standardised value moves any below-mean window in the wrong direction (and can drive IOB negative); Equation 16 is the only form of the perturbation that corresponds to a genuine 1.5*×* increase of the underlying physical quantity for every window, regardless of its value relative to the training mean. The perturbation is applied to the entire channel of the input window, so it represents a proportional increase in the model’s representation of on-board exposure rather than a single-timestep spike; the two perturbed forward passes share the batch’s other channels unchanged. Crucially, both penalties are applied as one-step forward-pass constraints on input-window perturbations during training; because the constraint acts on the training forward pass rather than on the recursive rollout, Section 4.3 tests empirically whether it transfers to the multi-step autoregressive setting, and Section 4 shows it does so only partially—consistent with a soft, non-architectural constraint that does not guarantee global monotonicity.

#### 3.6.6 Training protocol

Each patient-specific model is trained independently. A chronological 60/20/20 split produces non-overlapping training, validation, and held-out test sets. The split is *target-contained*: input windows and their prediction targets are constructed *within* each segment separately, so no window ever spans a split boundary and no future target can enter training. Because the splitting is target-contained, an explicit inter-segment embargo is not required to prevent target leakage; we verified on every participant that the last training target lies strictly inside the training segment and that all dose-response conditioning windows lie entirely inside the test segment. The StandardScaler is fitted exclusively on the training portion and then applied to validation and test, so no distributional information leaks across segments. Pharmacodynamic on-board states (insulin-on-board, carbohydrate-on-board) are allowed to carry their decayed history across segment boundaries, since a patient’s past insulin and carbohydrate exposure is legitimately known at inference time; only future glucose is withheld. The held-out test segment (the final 20% of each participant’s record) is reserved for all rollout, ablation, and dose-response evaluations reported in Section 4, while the validation segment is used only for checkpoint selection during training. Because the Manchester test segment was examined more than once during protocol development, the independent OhioT1DM cohort—processed and evaluated only after the protocol was frozen—serves as the clean confirmation cohort for every headline finding. The Adam optimiser (36) with learning rate 10*^−^*^3^ is used for 40 epochs with batch size 256. The best checkpoint (lowest validation RMSE across all 40 epochs) is saved; no early stopping is used. Each configuration was trained independently under five initialisation seeds (42–46); all other training settings were held fixed across seeds. **Table 6** summarises all hyperparameters.

**Table 6:** Hyperparameter summary for the core physiology-guided LSTM (full core configuration).

Component	Parameter	Value
Input sequence	Length *L* (SEQ_LEN)	72 steps (360 min)
Feature dimension	Core/Enriched	8/11
LSTM layers	num_layers	2
LSTM hidden units	hidden_size	128
Dropout	rate	0.20
Optimiser	—	Adam
Learning rate	LR	$1\times 10^{-3}$
Batch size	BATCH_SIZE	256
Training epochs	EPOCHS	40
Chronological split	train/val/test	60%/20%/20%
IOB decay constant	$\tau_{\text{ins}}$	180 min
COB decay constant	$\tau_{\text{carbs}}$	120 min
CGM masking probability	$p_{\text{mask}}$	0.60
Masking application rate	$p_{\text{apply}}$	0.40
Monotonicity penalty weight	λ	0.10
IOB/COB perturbation factor	—	1.50×
Evaluation horizons	—	5, 30, 60, 120 min
Rollout stride	STRIDE	6 steps (30 min)
Random seeds	—	42–46 (five independent initialisations)

Hyperparameters were set in two ways, and no value was tuned on any test set. The architecture and pharmacodynamic constants were specified *a priori* from prior literature and the data’s sampling characteristics: the 360-minute input window, two-layer hidden-128 LSTM, and Adam settings follow common CGM-forecasting practice (22), and the time constants ( $\tau_{\text{ins}}=180$, $\tau_{\text{carbs}}=120$ min) follow population pharmacodynamic approximations (33, 34). The regularisation settings ( $p_{\text{mask}}=0.60$, λ=0.10, 1.5× perturbation) were selected once on the Manchester *validation* segment (by validation RMSE), frozen before the Manchester test results reported here, and applied unchanged to the Ohio confirmation cohort. Because the qualitative findings (Section 4) reproduced across two cohorts and five seeds under this single frozen configuration, they do not depend on cohort-specific tuning, and a sensitivity analysis over the monotonicity weight λ (Section 4) confirms the accuracy–plausibility trade-off is stable around the frozen value

### 3.7 Model classes and ablation design

The evaluated model classes were designed to separate forecasting accuracy from intervention responsiveness:

1. Naive persistence: a non-learning zero-order-hold baseline that predicts the last observed glucose value at every horizon, ${\hat{ɡ}}_{t+h}={ɡ}_{t}$ for all *h*; by construction it cannot represent any response to insulin or carbohydrate.
2. Ridge autoregression (RidgeAR): a low-complexity, non-neural learning baseline—an *ℓ*_2_-regularised linear regression on the same flattened 72-step feature window, trained per participant on the identical split and evaluated under the same autoregressive rollout—included so that the comparison is not merely a neural network against last-value persistence.
3. Vanilla LSTM: a patient-specific recurrent model trained without explicit physiological constraints ( $p_{\text{mask}}=0$, *λ* = 0, IOB/COB features retained).
4. NoMask: a physiology-guided model with stochastic glucose masking removed ( $p_{\text{mask}}=0$, *λ* = 0.10).
5. NoMono: a model with directional monotonicity constraints removed ( $p_{\text{mask}}=0.60$, *λ* = 0).
6. NoIOB: pharmacodynamic ablation replacing the pre-computed IOB*_t_*with raw bolus dose; all other components retained.
7. NoCOB: pharmacodynamic ablation replacing the pre-computed COB*_t_*with raw meal carbohydrates; all other components retained.
8. Full Core: the complete physiology-guided model with IOB, COB, stochastic glucose masking, and double-monotonicity regularisation (Equations 3–15).

The ablation design is intentionally conservative. It does not claim that any single constraint is universally beneficial. Instead, it asks whether removing constraints changes the balance between observational accuracy and response to clinically motivated perturbations.

### 3.8 Forecasting and rollout protocol

One-step evaluation on the held-out test segment was conducted using training-only preprocessing and chronological separation. Multi-horizon evaluation used autoregressive rollout at 5, 30, 60, and 120 minutes. The four horizons were selected to span immediate, short-term, medium-term, and extended digital-twin forecasting regimes. The 5-minute horizon corresponds to one CGM sampling step and primarily tests local continuity. The 30-minute horizon reflects a commonly reported short-term glucose prediction interval in the glucose-forecasting literature. The 60-minute horizon provides a medium-range window in which insulin and carbohydrate effects begin to alter trajectory shape. The 120-minute horizon was included as a demanding evaluation of autoregressive accumulation, because a model intended for scenario simulation must remain behaviourally plausible beyond one-step prediction. In autoregressive rollout, the model’s predicted glucose is fed back as an input for subsequent prediction steps, making accumulated error visible. This is more demanding than one-step test evaluation and closer to the intended use of a digital twin for forward scenario simulation.

Because the paper’s thesis is that a single accuracy number is insufficient, forecasting performance is characterised on a small panel rather than by RMSE alone: RMSE (the primary accuracy metric, comparable to the forecasting literature), mean absolute error (MAE, less sensitive to large excursions), and mean signed error (bias, indicating systematic over- or under-prediction). This multidimensional accuracy view is reported so that the separation being demonstrated is between *forecasting accuracy as a whole* and treatment-response plausibility, not merely between one chosen error metric and one chosen plausibility metric. To address the exposure-bias concern (47)—that one-step training may itself disadvantage the neural models under multi-step rollout—we additionally fine-tune the accuracy-leading configuration with a genuine multi-step rollout loss (feeding the model its own predictions during training, in the spirit of scheduled sampling) and re-evaluate it under the same rollout protocol, testing whether the persistence baseline still leads at the longer horizons.

### 3.9 Insulin and carbohydrate dose-response audits and treatment-response plausibility

#### 3.9.1 Insulin audit

Treatment-response plausibility is evaluated with a graded insulin dose-response audit applied to every Core participant in both cohorts (Manchester *N* = 13, Ohio *N* = 12). We emphasise that this audit is a structured perturbation of the model’s learned insulin-exposure representation (its IOB*_t_*input channel), not a simulated administration of insulin governed by a physiological pharmacokinetic/pharmacodynamic model; the decay applied to the perturbation follows the same population-level exposure-proxy recursion used at training time (Section 3.4), not a patient-calibrated PK/PD model. The audit therefore asks a narrower, model-diagnostic question—does the candidate’s output move in the correct *direction* when its insulin-exposure input is increased—rather than certifying that its quantitative response magnitude or time-course is physiologically accurate. For each participant we draw 10 randomly sampled conditioning windows from the held-out test segment (fixed sampling seed = 42) and, from each window, run a 24-step (2-hour)autoregressive rollout at five insulin doses (+2, +4, +6, +10, +15 U) added to insulin-on-board at the first rollout step and decaying thereafter by the training-time recursion (*τ*_ins_ = 180 min). This yields *N ×* 10 *×* 5 trajectories per configuration per cohort.

For each dose we record the two-hour glucose change within the perturbed trajectory, Δ_abs_ (the change from the start to the end of the perturbed rollout). We deliberately audit Δ_abs_ rather than the difference against a no-dose counterfactual, because a strongly mean-reverting model can make the counterfactual-referenced quantity misleading at low doses; as a reviewer-requested robustness check, we additionally computed the matched no-intervention-referenced quantity from the identical conditioning windows and confirmed it yields the same conclusions (**Supplementary Table S10**). Aggregation proceeds in a fixed order: for a given participant, configuration, and seed, Δ_abs_ is first averaged *across the 10 conditioning windows* separately at each of the five doses, giving one mean response per dose; the dose-response slope is then fit by ordinary least squares on these five window-averaged, per-dose means (not by fitting a slope within each window and averaging the resulting slopes). From the five per-dose values we summarise each participant’s insulin response with three interpretable quantities: (i) the *dose-response slope*, the ordinary-least-squares slope of Δ_abs_ on dose (mg/dL per unit; directionally plausible when negative). A more negative slope indicates a stronger *relative* glucose-lowering response among the candidates compared here, under this specific perturbation protocol; it is not evidence that the magnitude of that response matches a real patient’s pharmacological sensitivity to insulin, which this audit is not designed to establish. (ii) a *sign-correctness* score, the fraction of doses whose mean response lowers glucose; and (iii) a *monotonicity* score, one minus the fraction of consecutive dose transitions in which the response moves in the wrong (glucose-raising) direction. The slope is the primary plausibility endpoint; sign-correctness and monotonicity are secondarydescriptors reported alongside it.

#### 3.9.2 Carbohydrate audit

The carbohydrate audit mirrors the insulin audit’s design with the perturbed channel and its physiologically expected direction reversed. For each participant we reuse the same 10 conditioning windows per cohort (fixed sampling seed = 42) and, from each window, run the same 24-step (2-hour) autoregressive rollout at four carbohydrate doses (+15, +30, +60, +90 g), applied as an impulse to carbohydrate-on-board at the first rollout step and decaying thereafter by the training-time recursion ( $\tau_{\text{carbs}}=120$ min); insulin-on-board is left to decay undisturbed over the same window. This yields *N ×*10*×*4 trajectories per configuration per cohort, evaluated for the three primary configurations (Full Core, Vanilla, NoMono). We treat the carbohydrate audit as supportive and exploratory relative to the insulin audit, which is the paper’s primary treatment-response endpoint: the carbohydrate endpoint is a discrete violation count over only three dose transitions, uses a single fixed $\tau_{hrmcarbs}$ that does not account for meal-composition variability (Limitations), and is not paired with a continuous dose-response slope of its own.

For each dose we record Δ_abs_ as in the insulin audit. Because a correctly-behaving twin should predict *more* carbohydrate to raise, not lower, glucose, we summarise each participant’s response with a *monotonicity-violation count*: the number of consecutive dose transitions (out of 3, since four doses yield three transitions) in which the mean response fails to increase with dose. This violation count, rather than a slope, is reported as the primary carbohydrate-audit statistic because the four-dose carbohydrate curve was pre-specified in the protocol as a directional pass/fail check; the analogous slope is not the primary endpoint here. **Supplementary Table S4** reports cohort-level mean violation counts for Full Core, Vanilla, and NoMono across all five seeds, together with the corresponding Friedman test (Section 4.3).

### 3.10 Multi-seed stability

Because patient-specific LSTMs are trained on small records, a single random initialisation could produce an unstable ranking. Every configuration in both cohorts is therefore trained under five independent seeds (42–46), holding all other settings fixed, giving 5*×*(6 neural configurations)*×* (13 + 12 patients) = 750 trained models plus the non-learning persistence baseline. All headline results are reported as the mean across seeds, and ordering claims are accompanied by the number of seeds (out of five) in which the ordering holds.

### 3.11 Independent replication cohort and protocol

OhioT1DM XML records were converted to the identical processed schema used for Manchester (5-minute regular grid; insulin-on-board and carbohydrate-on-board computed by the same exponential recursions with $\tau_{\text{ins}}=180$ and $\tau_{\text{carbs}}=120$ min). The two official Ohio files per participant were concatenated and re-split by the same chronological 60/20/20 rule used throughout, rather than using the official train/test split, so that both cohorts run one protocol and the comparison is not confounded by differing evaluation regimes; the aim is to test protocol generalisation, not to compete on a specific forecasting benchmark. The six Core neural configurations, the persistence baseline, the rollout protocol, the dose-response audit, and the multi-seed procedure are all applied unchanged. The single deliberate omission is the Enriched configuration: Ohio’s physiological-band streams are different modalities from Manchester’s sleep timeline, so a Core-versus-Enriched contrast on Ohio would not satisfy the cohort-fair definition. Two Ohio participants from the 2020 wave carry no heart-rate stream; this is irrelevant to the Core feature set used here.

### 3.12 Reproducibility package

Code and archived research artefacts are available at https://github.com/ameen-banjar/T1DUJxUoM (tagged release v5.0.0) and archived on Zenodo, doi:10.5281/zenodo.21794825 (9). The public package provides the model implementation, corrected perturbation logic, configuration files, regression tests, and an executable synthetic demonstration. The University of Manchester T1D-UOM dataset is openly available (published open-access under a CC BY 4.0 licence on Zenodo) (31); reproducing the exact patient-level outputs additionally requires the study-specific preprocessing and analysis workflow documented with the package. The OhioT1DM dataset (39) is not openly available and requires a separate data-use agreement with its maintainers.

## 4 Results

All results are reported on both cohorts (Manchester *N* = 13, OhioT1DM *N* = 12) with the participant as the unit of analysis and the five seeds treated as within-participant technical replication; unless stated otherwise, values are means across participants and seeds. We first show that neither contextual enrichment nor neural complexity improves forecasting accuracy, then that non-learning baselines lead at long horizons, then that, within the pre-specified primary comparison, the physiology constraint nevertheless yields the strongest correctly-directed insulin response (with the fuller ablation picture, including NoMask, given in Section 4.3), and finally which of these findings generalise across the two cohorts.

### 4.1 Forecasting accuracy: added context and neural complexity do not help

#### Contextual enrichment (Manchester)

In the paired overlapping subgroup (*N* = 10), adding sleep and wearable streams did not improve one-step held-out accuracy: mean RMSE was 8.94 mg/dL for Core versus 10.37 mg/dL for Enriched (Wilcoxon signed-rank *W* = 0, *p* = 0.002), with Enriched at or above Core for 9 of 10 participants. This paired contrast, and the per-participant window counts that make it cohort-fair (**Supplementary Table S1**), is reported in full in the **Supplementary Material**; it is a secondary result and is not multi-seeded, since the headline analyses concern the Core configurations.

#### Neural complexity (both cohorts)

Across the six neural configurations, the physiology-free Vanilla LSTM had the lowest one-step RMSE in *both* cohorts (Manchester 7.00 *±* 2.40 mg/dL; Ohio 5.02 *±* 0.97 mg/dL), and the physiology-guided Full Core was the worst or near-worst (Manchester 8.86; Ohio 12.29). A Friedman test across configurations was highly significant in both cohorts (Manchester *χ*^2^(5*,N* = 13) = 43.37, *p* = 3.1 *×* 10*^−^*^8^, Kendall’s *W* = 0.67; Ohio *χ*^2^(5*,N* = 12) = 46.67, *p* = 6.6 *×* 10*^−^*^9^, *W* = 0.78), and Vanilla was the single lowest-RMSE configuration in all five seeds of both cohorts. The Vanilla-versus-Full-Core contrast was significant in each cohort (Manchester *W* = 0, Holm-corrected *p* = 7.3 *×* 10*^−^*^4^; Ohio *W* = 0, Holm-corrected *p* = 1.5 *×* 10*^−^*^3^); the remaining pre-specified pairwise contrasts for this and every other inferential endpoint (rollout RMSE at all four horizons, the carbohydrate violation count) are reported in full, with Holm-corrected *p*-values throughout, in **Supplementary Table S5**. Adding physiological structure therefore costs one-step accuracy, consistently and reproducibly (**Figure 1**).

**Figure 1:**
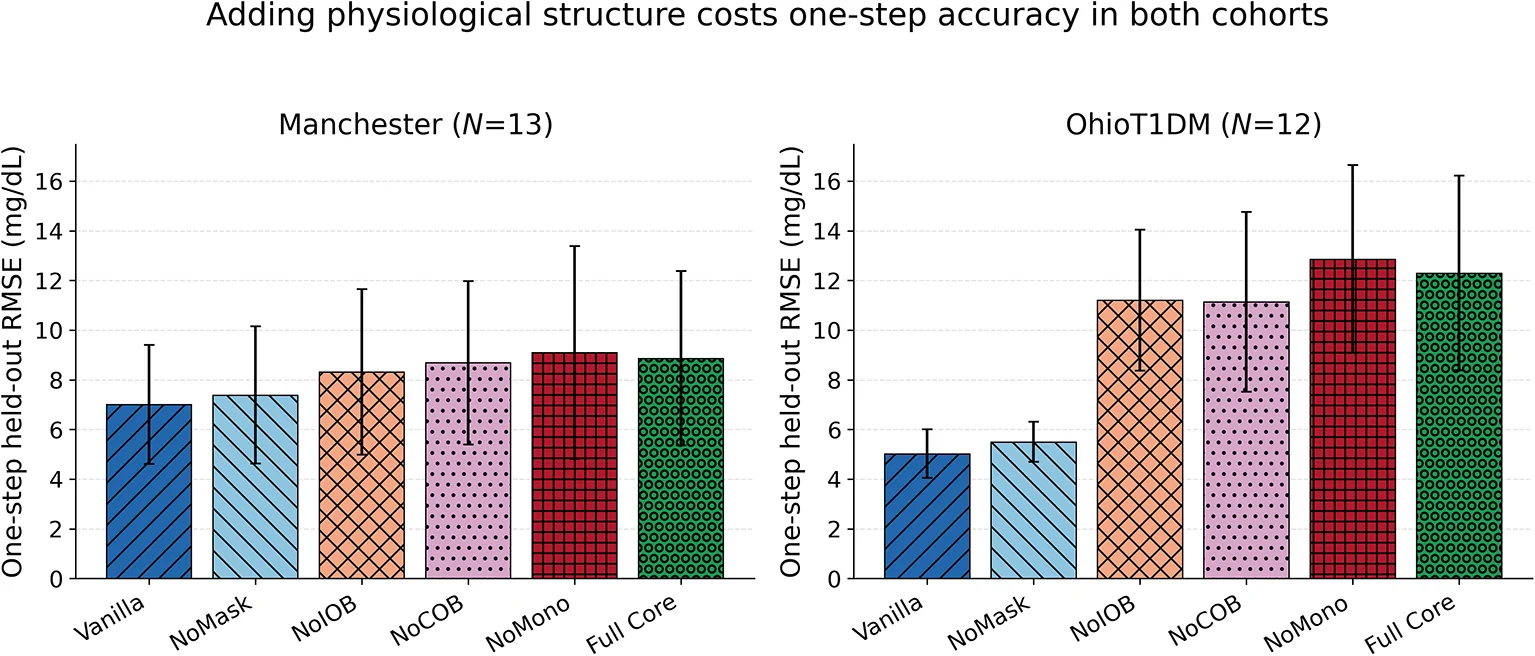
One-step held-out RMSE by configuration in both cohorts (mean *± *SD across participants and five seeds). The physiology-free Vanilla LSTM is the most accurate in both cohorts, and the physiology-guided Full Core is the worst or near-worst.

#### MAE and bias, with hierarchical bootstrap CIs

**Table 7** reports one-step MAE and mean signed error (bias) for the three configurations in the primary plausibility contrast, with 95% hierarchical bootstrap confidence intervals (participants resampled with replacement, seeds resampled within participant, 10,000 replicates), completing the multidimensional accuracy view promised in Section 3.6. MAE tracks RMSE closely and supports the same ordering (Vanilla most accurate, Full Core and NoMono similar and worse). Bias is small and its CI includes zero for every Manchester configuration and for Vanilla on Ohio, indicating no systematic over- or under-prediction. The one exception is Full Core on Ohio, which under-predicts by *−*2.04 mg/dL on average with a CI that excludes zero (*−*3.85 to *−*0.73); NoMono shows a smaller, borderline under-prediction on Ohio (*−*1.11, CI *−*2.59 to +0.07). This systematic Ohio under-prediction for the *λ >* 0 configurations is a modest but real accuracy cost of the physiological constraint on that cohort, distinct from the one-step RMSE gap already reported above. Because RMSE and MAE are pooled across the full glycaemic range, we additionally stratified one-step error by the true glucose value at the target step into hypoglycaemia (*<*70 mg/dL), target range (70–180 mg/dL), and hyperglycaemia (*>*180 mg/dL) for Full Core and Vanilla (**Supplementary Table S7**): in both cohorts, error is higher in the hypo- and hyperglycaemic strata than in the target range for both configurations, indicating that error is not uniform across the glycaemic range and is somewhat worse exactly where accurate forecasting matters most clinically.

**Table 7:** One-step held-out MAE and bias (mg/dL), cohort mean across participants and five seeds, with 95% hierarchical bootstrap confidence intervals (participant resampled with replacement, seed resampled within participant, 10,000 replicates).

Cohort	Manchester (*N* = 13)		OhioT1DM (*N* = 12)	
Configuration	MAE [95% CI]	Bias [95% CI]	MAE [95% CI]	Bias [95% CI]
Full Core	5.57 [4.38, 7.02]	+0.05 [*−*0.69, +0.92]	8.50 [6.94, 10.63]	*−*2.04 [*−*3.85, *−*0.73]*
Vanilla LSTM	4.13 [3.42, 4.98]	+0.02 [*−*0.50, +0.72]	2.98 [2.80, 3.15] *−*0.02	[*−*0.35, +0.32]
NoMono	5.48 [4.22, 7.01]	*−*0.00 [*−*0.66, +0.81]	8.70 [7.19, 10.40]	*−*1.11 [*−*2.59, +0.07]

Bias is mean signed error (prediction *−* actual); an interval excluding zero indicates a systematic over- or under-prediction.

*Bootstrap CI excludes zero: Full Core shows a small but systematic under-prediction on Ohio.

### 4.2 The RMSE paradox: non-learning baselines lead at the longer horizons

This finding is scoped to the architectures, training protocol, and evaluation regime used here: patient-specific recurrent (LSTM) models, trained one step ahead, evaluated under autoregressive multi-step rollout. It should not be read as a claim that persistence generally outperforms modern neural glucose-forecasting approaches; direct multi-horizon or population-pretrained architectures, which output all horizons at once rather than recursively re-feeding their own one-step predictions, were not evaluated here and may not be subject to the same autoregressive error accumulation (see Related Work and Limitations). **Table 8** reports multi-horizon autoregressive rollout RMSE for all configurations and both non-neural baselines. The naive persistence baseline,which by construction cannot represent any treatment effect, achieved lower RMSE than *every* neural configuration evaluated here at 60 and 120 minutes in both cohorts (e.g. 120-minute: Manchester 58.9 mg/dL vs 64.2 for Vanilla and 69.3 for Full Core; Ohio 58.4 vs 67.9 and 72.5). The non-neural Ridge-AR learning baseline was even lower than persistence on Manchester at, every horizon (52.6 mg/dL at 120 minutes), though it became unstable on Ohio at the longest horizon (89.1 mg/dL), illustrating that simple methods are competitive but not uniformly robust. Ranking candidates by forecasting error alone would therefore select a non-learning or linear baseline over any neural digital-twin configuration at the horizons most relevant to scenario simulation (**Figure 2**).

**Table 8:** Multi-horizon autoregressive rollout RMSE (mg/dL), cohort mean across participants and five seeds, for both cohorts.

Cohort	Manchester (*N* = 13)				OhioT1DM (*N* = 12)			
Configuration	5	30	60	120	5	30	60	120
Persistence	6.6	25.1	41.1	58.9	6.7	24.1	**39.5**	**58.4**
Ridge-AR	**6.2**	**23.2**	**36.9**	**52.6**	6.0	**21.7**	43.6	89.1
Vanilla LSTM	6.6	25.4	43.0	64.2	**5.9**	22.2	40.2	67.9
NoMask	6.9	27.7	49.0	75.0	6.3	28.4	54.5	88.1
NoIOB	7.4	27.6	44.6	66.0	11.6	32.2	51.6	72.2
NoCOB	8.0	27.8	45.1	67.0	12.2	33.0	50.9	72.2
NoMono	8.5	28.3	45.5	66.4	13.7	33.7	51.7	73.0
Full Core	8.2	28.2	46.3	69.3	13.2	33.7	52.1	72.5

Lower is better. Persistence is the non-learning baseline; Ridge-AR is the non-neural learning baseline. Bold marks the lowest value in each column.

**Figure 2:**
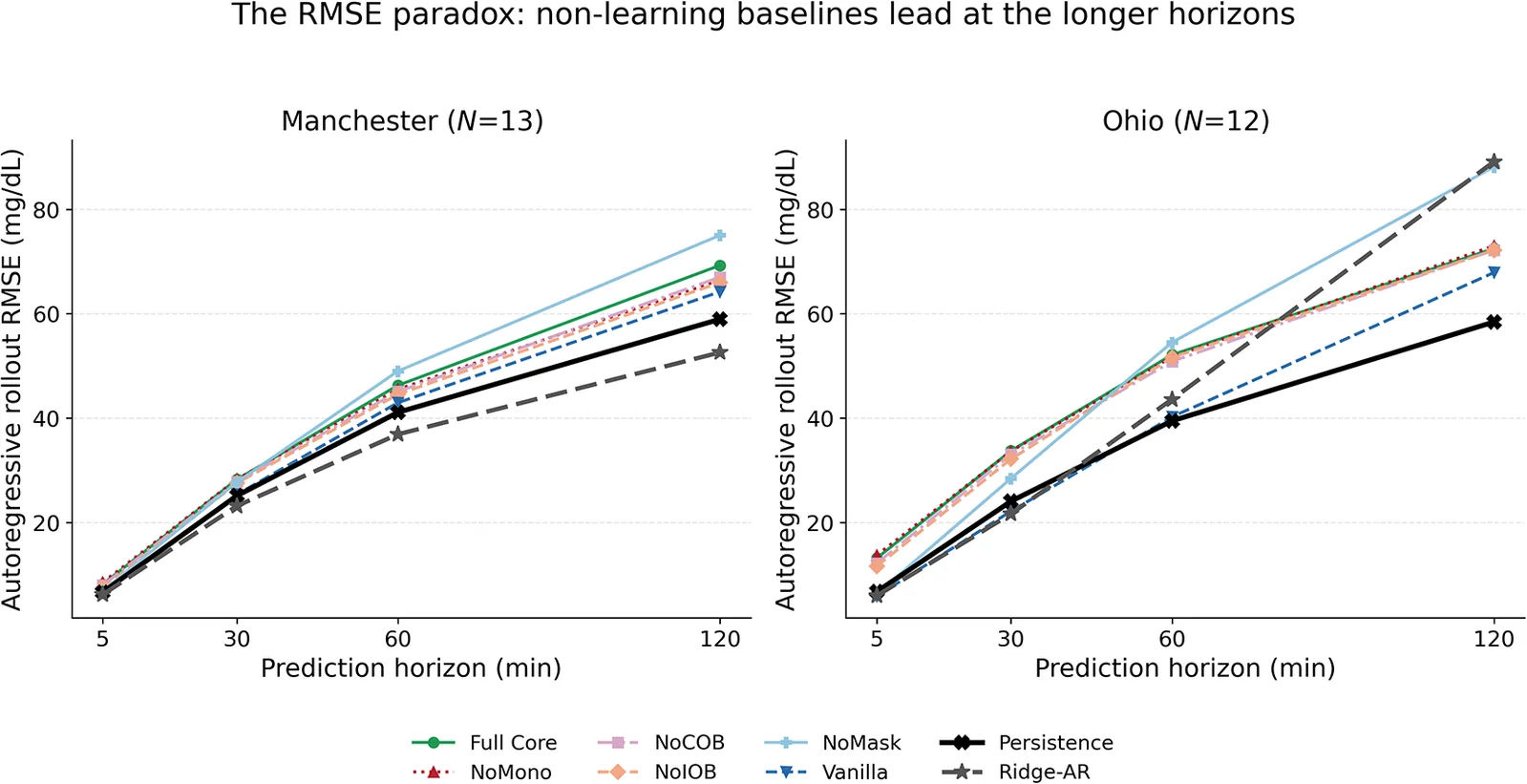
The RMSE paradox across horizons in both cohorts. Naive persistence (solid black) and the linear Ridge-AR baseline (dashed) have lower rollout RMSE than every neural configuration at 60 and 120 minutes on Manchester; on Ohio, persistence leads while Ridge-AR becomes unstable at the longest horizon.

The six-configuration difference underlying **Table 8** is significant at every horizon in both cohorts, surviving Holm correction across the four pre-specified horizons (**Supplementary Table S2**); thethree pairwise *post-hoc* contrasts at each horizon are in **Supplementary Table S5**.

This is not merely an exposure-bias artefact. Because the neural models were trainedone step ahead but evaluated under multi-step rollout, we tested whether the paradox is anartefact of that mismatch by fine-tuning the accuracy-leading Vanilla configuration with a genuine multi-step rollout loss (Section 3.6). Rollout-aware training did reduce the long-horizon error (120-minute Vanilla: Manchester 64.2 *→* 60.1; Ohio 67.9 *→* 63.5 mg/dL), confirming thatpart of the neural models’ disadvantage was exposure bias. However, persistence still matched or exceeded the rollout-aware model at 120 minutes in both cohorts (58.9 vs 60.1; 58.4 vs 63.5), and the two were comparable at 30–60 minutes. The RMSE paradox is thus real but bounded: low forecasting error remains achievable without learning treatment response, even after the training-evaluation mismatch is removed (**Figure 3**).

**Figure 3:**
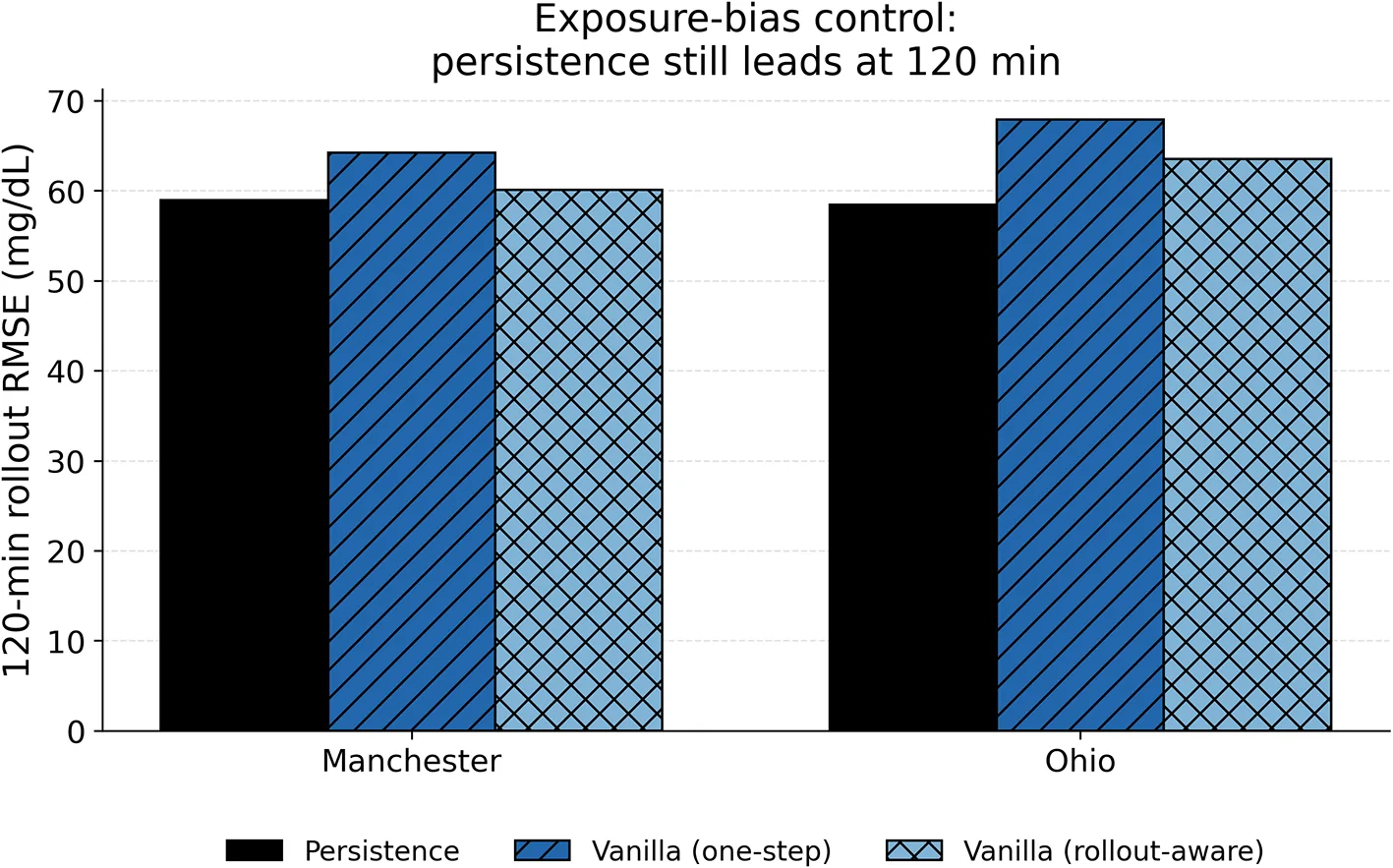
Exposure-bias control at 120 minutes. Rollout-aware fine-tuning lowers the neural model’s long-horizon error relative to one-step training, but naive persistence still matches or exceeds it in both cohorts.

### 4.3 Treatment-response plausibility: the constraint works, with a context-dependent margin

The graded insulin dose-response audit measures whether each configuration lowers predicted glucose as insulin dose increases. **Table 4** reports the three plausibility components; the dose-response slope (mg/dL per unit; negative is directionally plausible) is the primary endpoint. Within the pre-specified primary three-configuration comparison (Full Core, Vanilla, NoMono), Full Core produced the steepest negative slope in both cohorts (Manchester *−*5.24; Ohio *−*3.37), i.e. the strongest correctly-directed insulin response, and did so in all five seeds. As **Table 5** shows below, this ranking holds only within the pre-specified primary comparison: across all ablations, NoMask produced the steepest slope in both cohorts, and NoCOB was also slightly steeper than Full Core (Manchester *−*5.85 vs. *−*5.24; Ohio *−*3.57 vs. *−*3.37) despite retaining both the directional constraint and masking. Full Core is not the steepest-responding configuration overall. The unconstrained NoMono configuration produced a *positive* (wrong-signed) slope in both cohorts (+0.07 and +1.70), meaning that removing the directional penalty leaves a model whose predicted glucose, on average, rises with more insulin (**Figure 4**). This aggregate pattern is not driven by a few outlying participants: at the individual-participant level, the number of participants with a wrong-signed (positive) mean insulin slope is Full Core 1/13 (Manchester), 0/12 (Ohio); Vanilla 3/13, 3/12; NoMono 6/13, 5/12 (**Supplementary Figure S1** plots everyparticipant’s slope for all three configurations, both cohorts).

**Figure 4:**
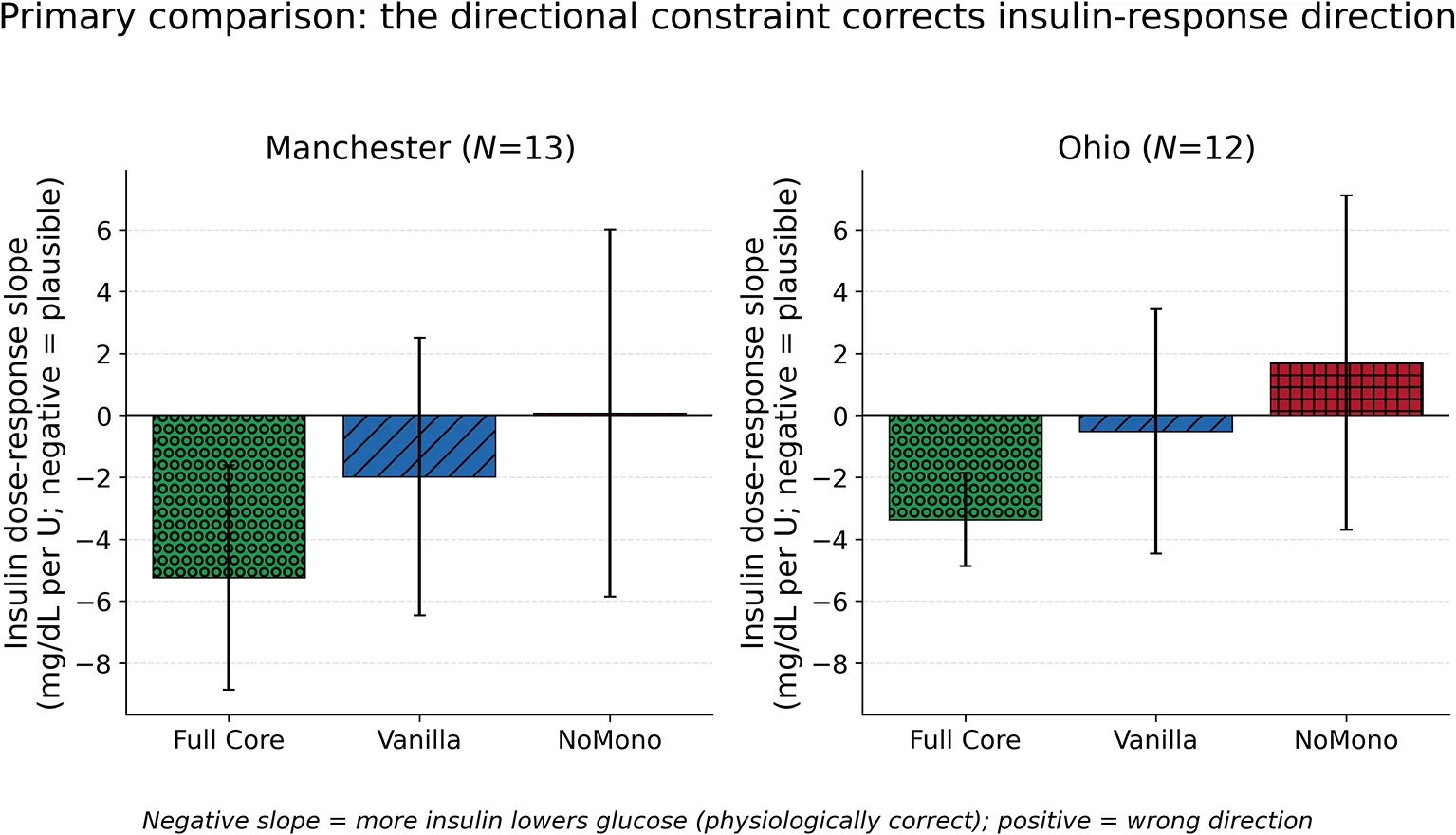
Primary comparison: the directional constraint corrects insulin-response direction (mean *± *SD across participants and five seeds). Within this pre-specified three-configuration comparison, Full Core has the steepest negative (physiologically correct) slope in both cohorts; NoMono is wrong-signed (positive) in both. The supplementary ablation NoMask (**Table 5**) has a steeper slope still; Full Core is not the steepest of every configuration tested.

**Figures 5**, **6** illustrate the cohort-level slope result as single-patient glucose trajectories underfour counterfactual scenarios, for one representative participant per cohort (Manchester 2301; OhioT1DM 563, chosen because its own NoMono slope, +1.70, matches the Ohio cohort mean almost exactly, so the trajectory is statistically typical rather than a selected best case). This is an illustrative single-window simulation, not a substitute for the participant-level statistics in **Table 4**. Under the dangerous-overbolus scenario (+15 U insulin, panel C), Full Core andVanilla both correctly trend the simulated glucose downward in both cohorts, while NoMono trends upward into the hyperglycaemic range in both cohorts despite the large insulin dose—the wrong-signed response the aggregate slope statistic quantifies.

**Figure 5:**
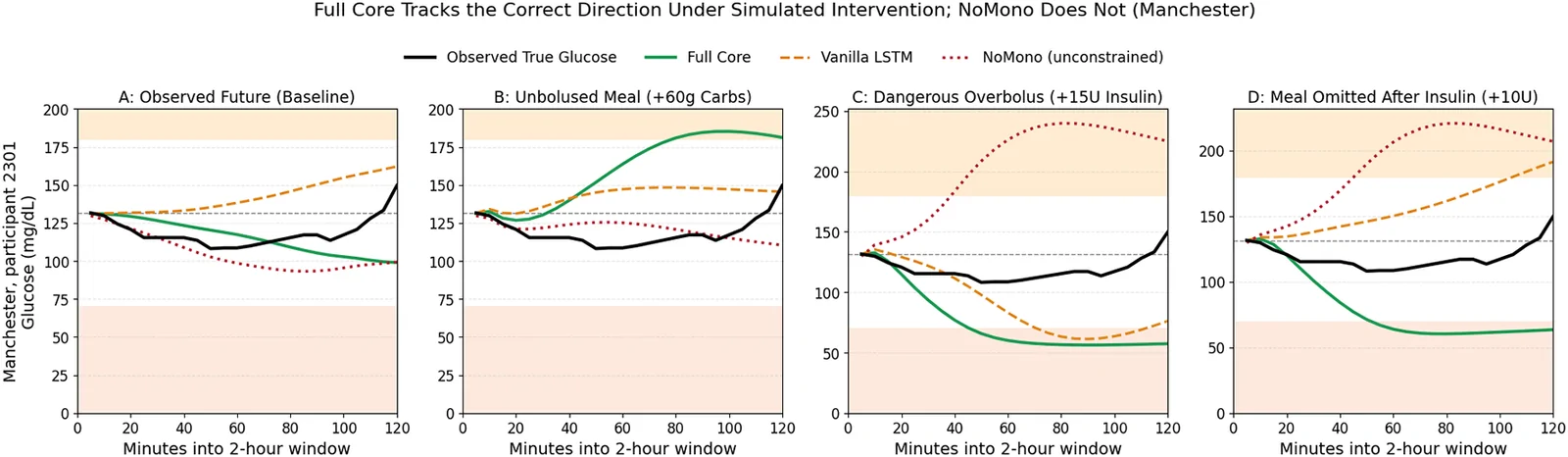
Counterfactual scenario simulation, Manchester participant 2301. Panels: **(A)** observed future, no intervention; **(B)** an unbolused 60g carbohydrate meal; **(C)** a dangerous +15U insulin overbolus; **(D)** a meal omitted after +10U insulin. Shaded bands mark hypoglycaemia (*<*70 mg/dL) and hyperglycaemia (*>*180 mg/dL). In the overbolus scenario **(C)**, only NoMono predicts glucose rising into hyperglycaemia despite the large insulin dose.

**Figure 6:**
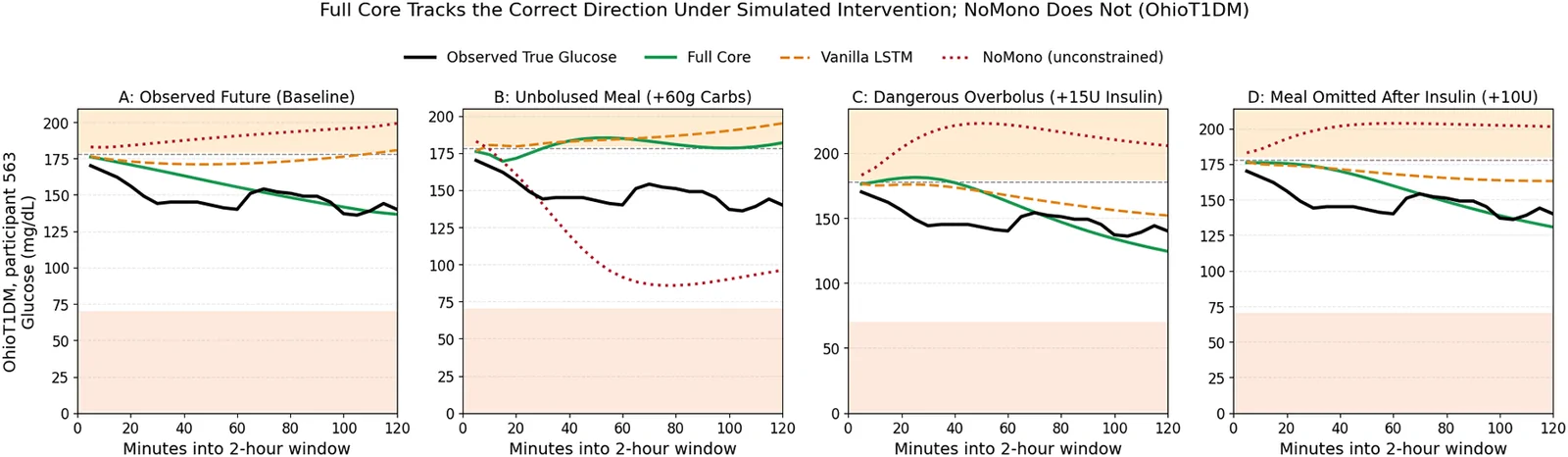
Counterfactual scenario simulation, OhioT1DM participant 563 (chosen as statistically typical for NoMono’s insulin slope in that cohort, not a selected best case). Panels as in **Figure 5**. The same pattern replicates independently: only NoMono predicts glucose rising into hyperglycaemia under the overbolus scenario **(C)**.

A Friedman test on the slopes was significant in both cohorts (Manchester *χ* (2*,N* = 13) = 11.23, *p* = 3.6 *×* 10*^−^*^3^, *W* = 0.43; Ohio *χ*^2^(2*,N* = 12) = 13.50, *p* = 1.2 *×* 10*^−^*^3^, *W* = 0.56). With Holm-corrected Wilcoxon *post-hoc* tests, Full Core had a significantly more plausible slope than NoMono in both cohorts, with a large matched-pairs rank-biserial effect size in each (Manchester *p* = 0.002, *r* = *−*0.71; Ohio *p* = 0.003, *r* = *−*0.69). The Full-Core-versus-Vanilla contrast, however, was cohort-dependent both in significance and in effect size: significant with a moderate effect on Ohio (*p* = 0.042, *r* = *−*0.41) but not on Manchester, where the effect size was negligible (*p* = 0.115, *r* = 0.01); see Section 4.4 for discussion of this pattern. The Vanilla-versus-NoMono contrast, a secondary comparison, was non-significant in Manchester (Holm-corrected *p* = 0.146, *r* = *−*0.21) but significant in Ohio (Holm-corrected *p* = 0.042, *r* = *−*0.41), consistent with removing the directional constraint being more clearly detrimental in the Ohio cohort. The analogous carbohydrate audit (Section 3.9.2), run across all five seeds in both cohorts for all three primary configurations, follows the same descriptive ordering as the insulin audit at the participant level (mean violations out of 3: Manchester Full Core 0.108, Vanilla 0.769, NoMono 0.938; Ohio Full Core 0.150, Vanilla 0.300, NoMono 0.900; cohort-level summary results in **Supplementary Table S4**). Friedman tests detected significant overall differences among the three configurations in both cohorts (Manchester *χ*^2^(2*, N* = 13) = 15.78, *p* = 4*×*10*^−^*^4^, *W* = 0.61; Ohio *χ*^2^(2*, N* = 12) = 10.14, *p* = 6.3 *×* 10, *W* = 0.42); the omnibus test does not by itself establish the specific ordering, so the three pairwise *post-hoc* contrasts (**Supplementary Table S5**) should be consulted for which differences are individually significant. Because the carbohydrate violation count is a discrete endpoint on {0,1,2,3} with many tied values and only 12–13 participants, we additionally re-tested the three primary contrasts with an exact sign-flip permutation test that makes no distributional or tie-handling assumption (**Supplementary Table S8a**); every contrast’s significance flag at *α* = 0.05 is unchanged from the Holm-corrected Wilcoxon result, so the carbohydrate finding is not an artefact of the asymptotic approximation.

Which physiological ingredient matters most for the insulin audit. **Table 5** extends the insulin dose-response audit to the three remaining ablations not shown in **Table 4**: NoMask (masking augmentation removed, monotonicity penalty retained), NoIOB (the explicit insulin-on-board state removed, replaced by the raw, non-decaying bolus event), and NoCOB (the carbohydrate-on-board state removed entirely). All three remain correctly-signed on average in both cohorts. NoCOB stays close to Full Core’s slope (Manchester *−*5.85 vs. *−*5.24; Ohio *−*3.57 vs. *−*3.37), whereas NoIOB is markedly weaker (Manchester *−*2.75; Ohio *−*1.52) — roughly half the magnitude of Full Core in both cohorts. This is the expected pattern mechanistically: IOB is the feature the insulin audit directly probes, so removing it and replacing it with a non-decaying raw event weakens the model’s capacity to represent a graded insulin effect far more than removing the (audit-irrelevant) COB state does. NoMask has the steepest slope of any configuration in both cohorts (Manchester *−*7.06; Ohio *−*7.27), consistent with the same patternalready noted for the primary contrast: removing the masking augmentation, independent of the monotonicity penalty, tends to strengthen the measured insulin response rather than weaken it. An exploratory (not pre-specified, uncorrected) paired comparison confirms NoMask is significantly steeper than Full Core in both cohorts (Manchester *p* = 0.0002; Ohio *p* = 0.0005; **Supplementary Table S8b**). Read together with **Table 8**, where NoMask is not the accuracy leader either, this indicates that within this ablation set the directional monotonicity penalty—not the combination of masking with the penalty that defines Full Core—is doing the work of improving directional responsiveness; Full Core should therefore be read as the pre-specified configuration that combines all physiological ingredients, not as the single best-performing configuration on either forecasting accuracy or response magnitude.

Sensitivity to the monotonicity weight *λ*. **Supplementary Table S3** varies the monotonicity weight across four values, λ∈{0, 0.05, 0.10, 0.20} (0 is NoMono, the directional penalty removed entirely), at fixed $p_{\text{mask}}=0.60$, evaluating one-step RMSE alongside the insulin slope, sign-correctness, and monotonicity.

One-step accuracy is essentially flat across *λ >* 0 in both cohorts (Manchester 8.86–9.07, Ohio 12.11–12.29 mg/dL), so the constraint’s accuracy cost is paid as soon as it is switched on and does not grow further with its strength. Sign-correctness and monotonicity both increase monotonically with *λ* in both cohorts.

The insulin slope itself increases monotonically with *λ* on Ohio (*−*2.49 *→ −*3.37 *→ −*4.38) but not on Manchester, where it strengthens from *λ* = 0.05 to 0.10 and then weakens slightly at *λ* = 0.20 (*−*4.81 *→ −*5.24 *→ −*4.90). The three Manchester values lie within one another’s standard deviation, so this is consistent with sampling noise around a plausibly still-monotonic trend rather than a true reversal; we report the non-monotonic point estimate rather than smoothing over it. In both cohorts, every *λ >* 0 value produces a materially stronger, moreconsistently signed, and more monotonic insulin response than *λ* = 0, supporting the stability of the accuracy–plausibility trade-off around the frozen operating point *λ* = 0.10 used throughout the paper.

### 4.4 What generalises, and what is context-dependent

Two principal findings replicated across both independent cohorts and all five seeds. The first is that *forecasting accuracy and treatment-response plausibility are separable*: within the pre-specified primary comparison, the accuracy leader (Vanilla) was not the plausibility leader (Full Core); across all ablations, NoMask produced the steepest insulin response of any configuration. Low forecasting error does not require learning treatment response, since non-learning and linear baselines lead at the longer horizons. The second is that *the directional constraint improves intervention responsiveness*: removing it (NoMono) leaves a wrong-signed insulin slope in both cohorts, whereas, within the pre-specified primary comparison, the physiology-guided model yields the strongest correctly-directed insulin response in both.

One finding was context-dependent: the advantage of the full physiology constraint *over the simpler Vanilla model* was significant on Ohio (Holm-corrected *p* = 0.042) but not on Manchester (Holm-corrected *p* = 0.115). We tested a signal-strength account of this pattern—that the constraint helps most where the mean insulin-on-board signal is weaker—by correlating mean insulin-on-board with the Full-Core-minus-Vanilla slope gap; the pooled Spearman correlation is *r* = *−*0.12 (*p* = 0.56, *n* = 25), and neither within-cohort correlation is significant (*r* = 0.12, *p* = 0.69 within Manchester; *r* = *−*0.31, *p* = 0.33 within Ohio). This does not support a signal-strength explanation. We therefore do not have a confirmed mechanistic account of why the Full-Core-versus-Vanilla contrast is cohort-dependent and report it as an open question rather than force a *post-hoc* explanation onto it.The practical implication is unchanged by this: the directional constraint is reliably beneficial relative to removing it entirely (Full Core significantly outperforms NoMono in both cohorts), while its marginal benefit over an unconstrained-but-otherwise-identical Vanilla model varies by cohort for reasons this study cannot yet identify.

## 5 Discussion

### 5.1 Why the RMSE paradox matters

The main finding is not that the physics-guided model is universally more accurate. It is not: across both cohorts and five seeds, the physiology-free Vanilla LSTM had lower one-step RMSE than Full Core, and under rollout a naive persistence baseline with no learned parameters was more accurate than every recurrent neural configuration evaluated here at 60 and 120 minutes (**Table 8**)—a scoped finding about patient-specific recurrent models under autoregressive rollout, not a general claim about neural glucose forecasting (see Related Work). Even after removing the training-evaluation mismatch with rollout-aware training, persistence still matched or exceeded the best neural model at the longest horizon. If RMSE were the only criterion, the optimal strategy would be to report the last observed glucose value—a trivial persistence baseline, not a model. Against this backdrop, the graded insulin dose-response audit tells the opposite story: among the primary configurations, the physiology-guided Full Core produced the steepest correctly-directed (glucose-lowering) response in both cohorts, whereas removing the directional constraint (NoMono) left a wrong-signed response in both (**Table 4**). The paper should therefore not be framed as an accuracy contest, but as an evaluation of two separable properties: forecast fit (where persistence, Ridge-AR, and Vanilla lead) and treatment-response plausibility (where Full Core leads significantly over NoMono in both cohorts, and significantly over Vanilla on Ohio but not on Manchester, for reasons not yet identified; Section 4.4).

This is the *autoregressive physiological insensitivity* introduced in Section 1: a configuration can minimise forecasting error by tracking recent CGM history while remaining insufficiently responsive to insulin and carbohydrate perturbations. It aligns with broader concerns about shortcut learning in deep neural networks (3). In T1D, the shortcut is especially plausible because CGM history is highly predictive and therapeutic events are observationally confounded. A bolus often follows high glucose, so a model can learn the wrong direction of association unless the training and evaluation protocol explicitly tests intervention response.

### 5.2 Architectural versus loss-based directional constraints

The present paper enforces directionally plausible insulin responsiveness through a loss-basedpenalty, $L_{\text{ins}}$, applied during training. Kushner et al. (23) addressed a closely related problem through an architectural constraint instead: their shallow network separates insulin-related inputs from non-insulin inputs in the first layer and restricts the insulin-related weights to act only in a glucose-lowering direction, so the desired sign of the insulin–glucose relationship is enforced by the network structure itself rather than by a training penalty. Both approaches target the same underlying failure mode—a model learning that insulin is associated with rising rather than falling glucose—but they make different trade-offs. An architectural constraint is enforced at every prediction step by construction, including under autoregressive rollout, whereas a loss-based penalty of the kind used here is enforced only at the training forward pass and, as Section 4.3 shows for $L_{\text{carbs}}$, may not transfer fully to multi-step rollout.

This contrast suggests a concrete direction for future work. The carbohydrate-responsiveness result (Section 4.3)—where Full Core has the fewest patient-level monotonicity violations but a non-zero violation rate in both cohorts—suggests that $L_{\text{carbs}}$ does transfer some directional signal to autoregressive rollout, but not perfectly. Retuning the loss weighting or the fixed $\tau_{\text{carbs}}=120$ minute decay constant discussed in the Limitations may help close this gap. Combining the present loss-based formulation with an architectural separation of carbohydrate-related inputs analogous to Kushner et al.’s treatment of insulin is also a plausible direction: a hybrid constraint, in which carbohydrate-on-board features are routed through a structurally restricted sub-network in addition to being penalised by $L_{\text{carbs}}$, could improve the consistency of the carbohydrate direction, and would be a natural focus for a rollout-aware double-monotonicity follow-up study.

### 5.3 Cohort size and the high-context feasibility framing

A central limitation is the small participant count (Manchester Core *N* = 13, OhioT1DM *N* = 12; 25 in total). This should be interpreted within the broader trade-off between multimodal richness and sample size in T1D datasets. Both cohorts provide detailed longitudinal streams across glucose, insulin, nutrition, activity, and sleep, making them suitable for testing whether neural digital-twin candidates respond plausibly to structured perturbations, and replication across two independent cohorts partly offsets the small per-cohort size. However, neither is sufficient to establish population-level generalisability or support clinical deployment claims. Each should therefore be interpreted as a *high-context feasibility cohort*: one whose value lies in stream density and cross-cohort replication rather than sample breadth. **Table 9** contextualises this within the T1D dataset landscape.

**Table 9:** Contextualisation of the University of Manchester dataset within the T1D dataset landscape.

Dataset	Value for this framework	Limitation	Replication note
UoM (this work)	High multimodal richness (glucose, insulin, nutrition, activity, sleep); suitable for scenario simulation responsiveness evaluation	Small *N*; feasibility cohort only	Primary dataset
OhioT1DM (39)	Rich contextual data (CGM, pump, self-report); multiple years available	Small *N* (*≈* 12 per phase); limited meal detail	Independent replication cohort used in this study
DiaTrend (40)	Larger *N* (*>*500); CGM and pump data	Limited behavioural context (no sleep/activity streams)	Suitable for observational accuracy benchmarking; limited for scenario simulation
DCLP3 (41)	Closed-loop trial context; well-controlled setting	Not designed for free-living multimodal analysis	Algorithm evaluation context; not open-loop multimodal
DIAMOND (42)	Large RCT (N = 158)	Not multimodal; no insulin pump data (MDI only)	Not suitable for this framework

The table identifies where external replication would be most feasible and what limitations would persist. UoM, University of Manchester.

The purpose of this work is therefore not to establish perfect per-patient physiological fidelity, but to provide a reproducible diagnostic protocol for identifying when low observational forecasting error fails to translate into directionally plausible scenario responsiveness. To test whether this protocol transfers beyond a single cohort, we repeated every core analysis on the independent OhioT1DM dataset (Section 3.11); because patient-specific models are retrained for each new participant, this is an independent-cohort replication of the modelling and evaluation *protocol*, not external validation of a fixed pre-trained model. Further replication on additional datasets such as DiaTrend (40) remains valuable.

### 5.4 Why the enriched cohort did not improve performance

The Enriched analysis should not be interpreted as evidence that sleep and wearable streams are irrelevant to glucose dynamics. Rather, it shows that adding additional modalities did not improve performance under the current cohort size, alignment, and modelling strategy: in the paired *N* = 10 subgroup, Enriched RMSE was at or above Core RMSE for 9 of 10 participants (8.94 vs. 10.37 mg/dL mean; Wilcoxon *p* = 0.002). The finding is consistent with a scenario in which the added streams are too sparse or temporally misaligned to provide reliableshort-horizon signal. Participant 2313 showed the largest divergence (Core: 6.50 mg/dL vs. Enriched: 9.65 mg/dL), and participant 2307, who had the shortest monitoring record (29 days), showed a similarly large gap (Core: 17.55 mg/dL vs. Enriched: 19.81 mg/dL), suggesting that sleep and wearable streams can actively harm model quality when they are incomplete. The single exception, participant 2304, showed a small Enriched advantage (9.37 vs. 8.97 mg/dL). Contextual variables may still be valuable when measured densely, temporally aligned to insulin and meals, and evaluated with sufficient participant numbers. The fair conclusion is that modality enrichment must be evaluated under paired designs rather than assumed beneficial by default.

### 5.5 Clinical caution

The dose-response audit intentionally uses graded and, at the highest dose, supra-physiologicalinsulin perturbations. However, the outputs are not clinical dosing recommendations; they aremodel-diagnostic results. A model that produces a large glucose-lowering response under a high insulin dose is not thereby shown to be safe; it is only shown to have learned a directionally plausible response to increased insulin exposure. Prospective clinical validation, risk analysis, uncertainty quantification, and regulatory review would be required before any clinical deployment claim.

### 5.6 Reproducibility implications

The repository is presented not merely as supplementary code but as a reproducibility object. The public package provides the model implementation, the corrected perturbation logic, configuration files, regression tests, and an executable synthetic end-to-end demonstration. Because the primary Manchester dataset is openly licensed (CC BY 4.0) and archived with a DOI, reproducing the exact patient-level paper outputs is possible by applying this same implementation to the downloaded Manchester data, following the protocol documentation; this additionally requires the study-specific preprocessing and analysis workflow described there, which is consistent withsoftware-citation principles and FAIR research-object expectations (10, 11).

## 6 Limitations

This study has several limitations. First, both cohorts are small (Manchester: 17 participants overall, 13 in the Core cohort, 10 in the paired Core-versus-Enriched subgroup; OhioT1DM: 12 participants), for 25 Core participants in total. Each should be interpreted as a high-context feasibility cohort whose value lies in multimodal stream density and cross-cohort replication rather than sample breadth; neither is sufficient to support population-level generalisability claims, and further replication on larger datasets such as DiaTrend (40) remains valuable. Second, patient-specific modelling reduces population heterogeneity but limits the ability to infer general performance across unseen individuals. Third, the dose-response audit tests directional responsiveness under structured perturbations but does not establish clinical validity; all outputs are model-diagnostic results, not treatment recommendations. Fourth, Enriched context maybe underpowered or insufficiently aligned rather than intrinsically unhelpful, as illustrated by participants 2313 and 2307 whose Enriched RMSE substantially exceeded Core RMSE. Fifth, the two cohorts differ in openness: the Manchester dataset is openly licensed (CC BY 4.0) so full reproduction is public, whereas OhioT1DM requires a data-use agreement, so the corresponding Ohio artefacts are provided as protocol and configuration rather than redistributed data.

Sixth, the dose-response audit conditions each rollout on 10 windows drawn at random (fixed sampling seed) from each participant’s held-out test segment, using the same windows for every configuration, and applies the same fixed insulin doses (+2 to +15 U) to every participant rather than doses personalised to each participant’s insulin sensitivity (ISF range: 14.0–72.7 mg/dL/U; **Table 2**). A given dose is therefore a larger relative intervention in insulin-sensitive participants. Relative to the empirical training-data distribution of observed bolus doses in each cohort (**Supplementary Table S6**), the audited doses of +2 to +10 U fall within the interquartile-to-90th-percentile range of observed single boluses in both cohorts, and the highest dose, +15 U, sits at approximately the 95th–96th percentile of observed boluses in both cohorts—at the upper tail of, but not outside, what models were actually trained on. We nonetheless treat +15 U as a diagnostic stress test rather than a realistic correction bolus, since it is deliberately chosen to be large relative to typical exposure. As an exploratory check of whether participant-level insulin sensitivity explains participant-level responsiveness, we correlated each Manchester participant’s ISF with their Full Core insulin dose-response slope (OhioT1DM’s public release has no per-participant ISF field, so this check is Manchester-only); the Spearman correlation is weak and non-significant (*ρ* = 0.16, *p* = 0.60, *n* = 13), which is consistent with treating the audit’s slope as measuring *directional* responsiveness rather than a quantity that scales with an individual’s known physiological insulin sensitivity. Personalised, ISF-scaled perturbations and an explicit assessment of response *magnitude* against clinical expectation remain natural extensions that this secondary analysis is not powered or designed to resolve.

Seventh, the current architecture does not propagate prediction uncertainty, so all dose-response and rollout outputs are point estimates. Future work should incorporate calibrated uncertainty intervals (e.g., conformal prediction (48), Monte Carlo dropout, or Bayesian recurrent networks) to make model-diagnostic outputs more informative for clinicians.

Eighth, the COB feature uses a fixed exponential decay constant ( $\tau_{\text{carbs}}=120$ min) following the population-average gastric-emptying approximation of Dalla Man et al. (34). Carbohydrate absorption in practice varies substantially with meal composition—from approximately 30–60 minutes for simple sugars to 180–240 minutes for complex or high-fat meals—and is further prolonged by gastroparesis, a complication prevalent in long-duration T1D (35). A single fixed *τ* may reduce the discriminability of the COB signal across diverse meal types and is a candidate explanation for why Full Core’s carbohydrate monotonicity-violation rate, while the lowest of the three primary configurations, is not zero (Section 4.3). Whether learnable or meal-type-stratified absorption parameters would improve carbohydrate responsiveness is an open question that motivates the rollout-aware constraint design proposed as future work.

Ninth, the rollout RMSE results in **Table 8** show that a naive persistence baseline outperforms every trained configuration, including Full Core, at 60 and 120 minutes in both cohorts. This is reported as a primary finding (Section 4) rather than only as a limitation, but it constrains how the dose-response results should be read: none of the trained configurations, including Full Core, should be described as accurate multi-step forecasters in absolute terms. Their value, if any, lies in directional responsiveness to perturbations that a non-learning persistence baseline cannot represent at all, since persistence by construction predicts no response to any perturbation.

Tenth, this study evaluates only patient-specific recurrent (LSTM) architectures trained one step ahead and evaluated under autoregressive multi-step rollout. It does not evaluate direct multi-horizon architectures (which output all horizons from a single forward pass rather than recursively re-feeding their own predictions), population-pretrained or transfer-learning models, or attention-based/Transformer forecasters (Related Work). Because these architectures are not necessarily subject to the same autoregressive error accumulation that produces the RMSE paradox reported here, the finding that non-learning baselines lead at longer rollout horizons should be read as scoped to the recurrent, patient-specific, autoregressive-rollout setting evaluated in this paper, not as a general claim about neural glucose forecasting.

## 7 Conclusion

This study shows that observational forecasting accuracy and directional treatment-response plausibility are separable properties of a T1D digital-twin candidate: low forecasting error does not guarantee a directionally sensitive response to insulin or carbohydrate perturbations. Both findings held on the Manchester development cohort and reproduced on the independent OhioT1DM confirmation cohort, across five seeds. An explicit double-monotonicity constraint materially changed directional sensitivity—removing it (NoMono) produced a wrong-signed insulin response in both cohorts—but including it (Full Core) did not make it the single steepest-responding configuration overall (NoMask, and marginally NoCOB, were steeper); its advantage was significant and consistent only against the unconstrained NoMono baseline, while its advantage over the simpler Vanilla LSTM was cohort-dependent (significant on Ohio, not on Manchester, for reasons we cannot yet explain; Section 4.4). We therefore state the constraint’s contribution modestly: it alters a candidate’s directional sensitivity to therapeutic perturbation; it does not guarantee superiority over every simpler alternative, and it does not by itself establish that a candidate is validated for any interventional use. The protocol proposed here is a reproducible model-diagnostic screening step, not a clinical decision system. It is not a claim that Full Core is an accurate forecaster—by RMSE it is the worst or near-worst of the six neural configurations at every horizon—nor that its dose-response audit establishes physiological validity rather than directional plausibility. Its value is narrower: forecasting accuracy alone cannot certify that a candidate twin behaves sensibly under a therapeutic perturbation, so the two properties should be screened separately before any candidate twin is considered for in-silico intervention use. Future work should pursue rollout-aware physiological constraints, quantitative (not only directional) validation against pharmacokinetic references, and uncertainty quantification, to move from behavioural screening toward stronger evidentiary claims.

## Data Availability

The original contributions presented in the study are included in the article/**Supplementary Material**. Further inquiries can be directed to the corresponding author.
