# Supplementary material for: Forecasting accuracy is not enough: safety-oriented evaluation of neural digital twins prior to in-silico insulin and carbohydrate intervention in type 1 diabetes
Source: Front Endocrinol (Lausanne). 2026 Oct 14;17:1958918. doi: 10.3389/fendo.2026.1958918 (PMC13714816; doi:10.3389/fendo.2026.1958918)
Supplement: Supplementary file 1 [file SupplementaryFile1.pdf]

# Supplementary Material

Forecasting Accuracy Is Not Enough: Safety-Oriented Evaluation of Neural Digital Twins Prior to In-Silico Insulin and Carbohydrate Intervention in Type 1 Diabetes

This document contains supplementary tables and figures referenced from the main manuscript as Supplementary Table S1–S10 and Supplementary Figure S1.

**Table S1:** Per-participant preprocessing quality control for both cohorts. Days: recording span; Samples: usable 5-min rows; Bolus/Meals: event counts; Train/Val/Test: valid input windows per split.

| Cohort | PID | Days | Samples | Bolus | Meals | Train | Val | Test |
|---|---|---|---|---|---|---|---|---|
| Manchester | 2301 | 109 | 31,680 | 549 | 252 | 18,936 | 6,264 | 6,264 |
| Manchester | 2302 | 169 | 48,809 | 175 | 215 | 29,213 | 9,690 | 9,690 |
| Manchester | 2304 | 116 | 33,529 | 179 | 202 | 20,045 | 6,634 | 6,634 |
| Manchester | 2305 | 63 | 18,431 | 130 | 94 | 10,986 | 3,614 | 3,615 |
| Manchester | 2306 | 102 | 29,527 | 423 | 365 | 17,644 | 5,833 | 5,834 |
| Manchester | 2307 | 29 | 8,535 | 292 | 129 | 5,049 | 1,635 | 1,635 |
| Manchester | 2308 | 106 | 30,623 | 389 | 242 | 18,301 | 6,053 | 6,053 |
| Manchester | 2309 | 85 | 24,651 | 286 | 200 | 14,718 | 4,858 | 4,859 |
| Manchester | 2313 | 99 | 28,676 | 258 | 163 | 17,133 | 5,663 | 5,664 |
| Manchester | 2314 | 91 | 26,320 | 313 | 481 | 15,720 | 5,192 | 5,192 |
| Manchester | 2401 | 91 | 26,492 | 241 | 485 | 15,823 | 5,226 | 5,227 |
| Manchester | 2403 | 116 | 33,567 | 321 | 181 | 20,068 | 6,641 | 6,642 |
| Manchester | 2405 | 98 | 28,419 | 289 | 255 | 16,979 | 5,612 | 5,612 |
| OhioT1DM | 540 | 56 | 13,824 | 335 | 84 | 8,222 | 2,693 | 2,693 |
| OhioT1DM | 544 | 54 | 13,496 | 149 | 166 | 8,025 | 2,627 | 2,628 |
| OhioT1DM | 552 | 49 | 9,587 | 276 | 67 | 5,680 | 1,845 | 1,846 |
| OhioT1DM | 559 | 51 | 13,607 | 159 | 165 | 8,092 | 2,649 | 2,650 |
| OhioT1DM | 563 | 54 | 14,804 | 398 | 145 | 8,810 | 2,889 | 2,889 |
| OhioT1DM | 567 | 53 | 12,723 | 272 | 27 | 7,561 | 2,473 | 2,473 |
| OhioT1DM | 570 | 50 | 13,887 | 356 | 160 | 8,260 | 2,705 | 2,706 |
| OhioT1DM | 575 | 54 | 14,856 | 200 | 271 | 8,841 | 2,899 | 2,900 |
| OhioT1DM | 584 | 56 | 15,178 | 269 | 108 | 9,034 | 2,964 | 2,964 |
| OhioT1DM | 588 | 55 | 15,503 | 213 | 249 | 9,229 | 3,029 | 3,029 |
| OhioT1DM | 591 | 54 | 13,761 | 271 | 227 | 8,184 | 2,680 | 2,681 |
| OhioT1DM | 596 | 57 | 13,787 | 210 | 247 | 8,200 | 2,685 | 2,686 |

**Table S2:** Per-horizon Friedman omnibus test across the six neural configurations for the multi-horizon rollout RMSE endpoint reported in Table 6 of the main manuscript, with Holm correction across the four horizons within each cohort. All eight tests remain significant after correction.

| Cohort | Horizon (min) | $\chi^2(5)$ | Raw $p$ | Holm $p$ | Kendall's $W$ |
|---|---|---|---|---|---|
| Manchester | 5 | 32.65 | $< 10^{-4}$ | $< 10^{-4}$ | 0.50 |
| Manchester | 30 | 22.19 | $4.8 \times 10^{-4}$ | $1.4 \times 10^{-3}$ | 0.34 |
| Manchester | 60 | 20.78 | $8.9 \times 10^{-4}$ | $1.8 \times 10^{-3}$ | 0.32 |
| Manchester | 120 | 17.53 | $3.6 \times 10^{-3}$ | $3.6 \times 10^{-3}$ | 0.27 |
| OhioT1DM | 5 | 46.29 | $< 10^{-7}$ | $< 10^{-7}$ | 0.77 |
| OhioT1DM | 30 | 28.38 | $3.1 \times 10^{-5}$ | $1.2 \times 10^{-4}$ | 0.47 |
| OhioT1DM | 60 | 20.71 | $9.2 \times 10^{-4}$ | $9.2 \times 10^{-4}$ | 0.35 |
| OhioT1DM | 120 | 22.24 | $4.7 \times 10^{-4}$ | $9.4 \times 10^{-4}$ | 0.37 |

**Table S3:** Sensitivity of one-step accuracy and insulin dose-response plausibility to the monotonicity weight $\lambda$, participant-level mean ($\pm$SD where applicable) across five seeds. $\lambda = 0$ (NoMono) removes the directional penalty entirely; $\lambda = 0.10$ (Full Core) is the frozen value used throughout the paper.

| | Manchester ($N = 13$) | | | | OhioT1DM ($N = 12$) | | | |
|---|---|---|---|---|---|---|---|---|
| $\lambda$ | One-step RMSE | Slope | Sign | Mono | One-step RMSE | Slope | Sign | Mono |
| 0 (NoMono) | 9.10±4.29 | +0.07±5.93 | 0.65 | 0.57 | 12.86±3.78 | +1.70±5.40 | 0.56 | 0.50 |
| 0.05 | 8.96±3.78 | −4.81±3.87 | 0.83 | 0.86 | 12.11±3.30 | −2.49±2.86 | 0.86 | 0.83 |
| 0.10 (Full Core) | 8.86±3.51 | −5.24±3.63 | 0.88 | 0.92 | 12.29±3.92 | −3.37±1.50 | 0.93 | 0.95 |
| 0.20 | 9.07±3.52 | −4.90±3.07 | 0.94 | 0.92 | 12.28±3.39 | −4.38±1.75 | 0.92 | 0.97 |

**Table S4:** Carbohydrate dose-response audit: cohort-level mean ($\pm$SD across participants) monotonicity-violation count (out of 3 dose transitions; lower is better) across five seeds, for the three primary configurations, both cohorts. The means follow the descriptive ordering Full Core < Vanilla < NoMono in both cohorts. Friedman tests detect a significant overall difference among the three configurations in both cohorts (Manchester $\chi^2(2, N = 13) = 15.78$, $p = 4 \times 10^{-4}$, Kendall's $W = 0.61$; Ohio $\chi^2(2, N = 12) = 10.14$, $p = 6.3 \times 10^{-3}$, $W = 0.42$); see Supplementary Table S5 for the pairwise post-hoc contrasts.

| Configuration | Manchester ($N = 13$) | OhioT1DM ($N = 12$) |
|---|---|---|
| Full Core | 0.108 ± 0.278 | 0.150 ± 0.243 |
| Vanilla LSTM | 0.769 ± 0.652 | 0.300 ± 0.501 |
| NoMono | 0.938 ± 0.984 | 0.900 ± 0.872 |

**Table S5:** Full pre-specified post-hoc reporting (Holm-corrected Wilcoxon signed-rank tests and matched-pairs rank-biserial effect size) for the three primary pairwise contrasts, across every inferential endpoint promised in the Methods *Planned tests* paragraph: one-step RMSE, multi-horizon rollout RMSE (all four horizons), the carbohydrate violation count, and the insulin dose-response slope (already reported in-text; included here for a single complete reference). Asterisk marks Holm-corrected $p < 0.05$.

| Endpoint | Cohort | Contrast | $W$ | Holm $p$ | $r$ |
|---|---|---|---|---|---|
| One-step RMSE | Manchester | FullCore vs Vanilla | 0.0 | 0.0007* | 1.00 |
| | Manchester | FullCore vs NoMono | 28.0 | 0.2439 | 0.78 |
| | Manchester | Vanilla vs NoMono | 0.0 | 0.0007* | -1.00 |

| Endpoint | Cohort | Contrast | $W$ | Holm $p$ | $r$ |
|---|---|---|---|---|---|
| | Ohio | FullCore vs Vanilla | 0.0 | 0.0015* | 1.00 |
| | Ohio | FullCore vs NoMono | 25.0 | 0.3013 | 0.28 |
| | Ohio | Vanilla vs NoMono | 0.0 | 0.0015* | -1.00 |
| Rollout RMSE (5 min) | Manchester | FullCore vs Vanilla | 3.0 | 0.0024* | 0.98 |
| | Manchester | FullCore vs NoMono | 33.0 | 0.4143 | 0.78 |
| | Manchester | Vanilla vs NoMono | 0.0 | 0.0007* | -1.00 |
| Rollout RMSE (30 min) | Manchester | FullCore vs Vanilla | 9.0 | 0.0161* | 0.98 |
| | Manchester | FullCore vs NoMono | 37.0 | 0.5879 | 0.67 |
| | Manchester | Vanilla vs NoMono | 6.0 | 0.0103* | -0.71 |
| Rollout RMSE (60 min) | Manchester | FullCore vs Vanilla | 17.0 | 0.0957 | 0.93 |
| | Manchester | FullCore vs NoMono | 28.0 | 0.2439 | 0.54 |
| | Manchester | Vanilla vs NoMono | 11.0 | 0.0403* | -0.71 |
| Rollout RMSE (120 min) | Manchester | FullCore vs Vanilla | 22.0 | 0.3296 | 0.87 |
| | Manchester | FullCore vs NoMono | 24.0 | 0.3296 | 0.87 |
| | Manchester | Vanilla vs NoMono | 33.0 | 0.4143 | 0.01 |
| Rollout RMSE (5 min) | Ohio | FullCore vs Vanilla | 0.0 | 0.0015* | 1.00 |
| | Ohio | FullCore vs NoMono | 25.0 | 0.3013 | 0.28 |
| | Ohio | Vanilla vs NoMono | 0.0 | 0.0015* | -1.00 |
| Rollout RMSE (30 min) | Ohio | FullCore vs Vanilla | 1.0 | 0.0020* | 0.97 |
| | Ohio | FullCore vs NoMono | 36.0 | 0.8501 | 0.28 |
| | Ohio | Vanilla vs NoMono | 0.0 | 0.0015* | -1.00 |
| Rollout RMSE (60 min) | Ohio | FullCore vs Vanilla | 3.0 | 0.0049* | 0.97 |
| | Ohio | FullCore vs NoMono | 39.0 | 1.0000 | 0.46 |
| | Ohio | Vanilla vs NoMono | 1.0 | 0.0029* | -0.69 |
| Rollout RMSE (120 min) | Ohio | FullCore vs Vanilla | 22.0 | 0.4072 | 0.74 |
| | Ohio | FullCore vs NoMono | 37.0 | 0.9097 | 0.62 |
| | Ohio | Vanilla vs NoMono | 16.0 | 0.2314 | 0.08 |
| Carbohydrate violation count | Manchester | FullCore vs Vanilla | 0.0 | 0.0039* | -1.00 |
| | Manchester | FullCore vs NoMono | 0.0 | 0.0015* | -1.00 |
| | Manchester | Vanilla vs NoMono | 32.5 | 0.9941 | 0.55 |
| | Ohio | FullCore vs Vanilla | 6.0 | 0.8750 | 0.60 |
| | Ohio | FullCore vs NoMono | 0.0 | 0.0234* | -1.00 |
| | Ohio | Vanilla vs NoMono | 3.0 | 0.0781 | -0.56 |
| Insulin dose-response slope | Manchester | FullCore vs Vanilla | 18.0 | 0.1147 | 0.01 |
| | Manchester | FullCore vs NoMono | 1.0 | 0.0015* | -0.71 |
| | Manchester | Vanilla vs NoMono | 24.0 | 0.1465 | -0.21 |
| | Ohio | FullCore vs Vanilla | 10.0 | 0.0420* | -0.41 |
| | Ohio | FullCore vs NoMono | 1.0 | 0.0029* | -0.69 |
| | Ohio | Vanilla vs NoMono | 13.0 | 0.0425* | -0.41 |

**Table S6:** Empirical training-data distributions of bolus insulin dose, meal carbohydrate size, and the two on-board exposure proxies (IOB, COB), by cohort, and the percentile rank of each audited perturbation dose within the corresponding observed event distribution. All quantities are computed from the same processed training data used to fit the models (no held-out or test-only restriction), pooled across all Core participants in each cohort. "Nonzero events" restricts to timesteps with an actual bolus or meal, not the many zero-valued 5-minute bins between events.

| Cohort | Quantity | *N* | Median | Q25 | Q75 | P90 | P95 (Max) |
|---|---|---|---|---|---|---|---|
| Manchester | Bolus dose, U (nonzero events) | 3845 | 4.25 | 2.00 | 7.00 | 10.00 | 14.00 (68.0) |
| Manchester | Meal carbohydrate, g (nonzero events) | 3264 | 43.0 | 24.0 | 66.0 | 95.0 | 118.0 (283.2) |
| Manchester | Insulin-on-board, U (all timesteps) | 369,259 | 0.74 | 0.00 | 2.68 | 5.32 | 7.67 (78.1) |
| Manchester | Carbohydrate-on-board, g (all timesteps) | 369,259 | 1.65 | 0.01 | 12.68 | 33.63 | 50.30 (287.9) |
| OhioT1DM | Bolus dose, U (nonzero events) | 3108 | 5.00 | 2.80 | 8.72 | 12.10 | 14.80 (38.4) |
| OhioT1DM | Meal carbohydrate, g (nonzero events) | 1916 | 40.0 | 22.0 | 60.0 | 90.0 | 120.0 (450.0) |
| OhioT1DM | Insulin-on-board, U (all timesteps) | 165,013 | 2.79 | 1.12 | 5.86 | 10.37 | 13.82 (51.1) |
| OhioT1DM | Carbohydrate-on-board, g (all timesteps) | 165,013 | 5.29 | 0.53 | 18.28 | 36.83 | 51.47 (452.1) |

**Audited perturbation doses relative to the observed bolus/meal distributions above:**

| Perturbation | Manchester percentile | OhioT1DM percentile |
|---|---|---|
| Insulin +2 U | 24.9th | 15.0th |
| Insulin +4 U | 46.5th | 39.7th |
| Insulin +6 U | 67.1th | 57.5th |
| Insulin +10 U | 88.7th | 84.3th |
| Insulin +15 U | 95.9th | 95.4th |
| Carbohydrate +15 g | 15.4th | 13.5th |
| Carbohydrate +30 g | 36.0th | 36.6th |
| Carbohydrate +60 g | 70.5th | 76.0th |
| Carbohydrate +90 g | 87.9th | 89.9th |

**Table S7:** One-step forecasting error stratified by the true glucose value at the target step, for the three clinically relevant glycaemic ranges. MAE and RMSE (mg/dL) are pooled across all test-segment predictions for all participants and (for FullCore/Vanilla) all five seeds. *Naive one-step persistence is included for completeness but is not a comparable baseline here*: because CGM changes little over a single 5-minute step, one-step persistence is known to be a very strong (near-tautological) short-horizon predictor for any continuous physiological signal, and this is unrelated to the multi-step rollout RMSE paradox reported as the paper's primary accuracy finding (Results, Section 4 of the main manuscript), where persistence's advantage emerges only after autoregressive accumulation. Within the two trained neural configurations, error is higher in both hypo- and hyperglycaemia than in the target range for both cohorts, i.e. forecasting error is not uniform across the glycaemic range.

| Cohort | Configuration | Hypoglycaemia (<70 mg/dL) MAE / RMSE | Target (70–180 mg/dL) MAE / RMSE | Hyperglycaemia (>180 mg/dL) MAE / RMSE |
|---|---|---|---|---|
| Manchester | Full Core | 6.42 / 10.07 | 4.34 / 7.58 | 6.75 / 11.31 |
| Manchester | Vanilla | 4.35 / 7.55 | 3.63 / 6.65 | 5.19 / 9.13 |
| Manchester | Persistence* | 2.52 / 6.02 | 3.31 / 6.99 | 4.40 / 8.88 |
| OhioT1DM | Full Core | 8.41 / 10.79 | 8.08 / 12.59 | 9.00 / 13.64 |
| OhioT1DM | Vanilla | 2.94 / 4.15 | 2.74 / 4.68 | 3.38 / 5.86 |
| OhioT1DM | Persistence* | 2.42 / 3.84 | 3.53 / 5.54 | 4.27 / 6.82 |

*See caveat above: one-step persistence is not part of the pre-specified one-step comparison in the main text and should not be read as extending the rollout RMSE paradox to one-step prediction.

**Table S8:** Two exploratory/robustness analyses, reported separately from the pre-specified primary comparisons in Table S5 and not subjected to a shared multiplicity correction across the two. **(a) Carbohydrate violation-count robustness check:** an exact sign-flip permutation test (no distributional or tie-handling assumption; appropriate given the discrete 0–3 endpoint and $N = 12$–$13$) re-testing the three primary contrasts already reported with Holm-corrected Wilcoxon signed-rank tests in Table S5. Every contrast's significance flag at $\alpha = 0.05$ is unchanged from the Wilcoxon result, confirming the carbohydrate finding is not an artefact of the asymptotic approximation. **(b) Full Core vs. NoMask (insulin slope), exploratory:** NoMask is not part of the pre-specified primary three-configuration comparison; this paired contrast is motivated post hoc by NoMask having the steepest slope of any configuration (main manuscript, "Which physiological ingredient matters most for the insulin audit") and is reported here as hypothesis-generating, not confirmatory.

**(a) Carbohydrate violation count: exact permutation test vs. Wilcoxon (Table S5)**

| Cohort | Contrast | Mean diff. | Exact $p$ | Exact Holm $p$ |
|---|---|---|---|---|
| Manchester | FullCore vs Vanilla | −0.66 | 0.0020 | 0.0039* |
| Manchester | FullCore vs NoMono | −0.83 | 0.0005 | 0.0015* |
| Manchester | Vanilla vs NoMono | −0.17 | 0.5293 | 0.5293 |
| OhioT1DM | FullCore vs Vanilla | −0.15 | 0.5000 | 0.5000 |
| OhioT1DM | FullCore vs NoMono | −0.75 | 0.0078 | 0.0234* |
| OhioT1DM | Vanilla vs NoMono | −0.60 | 0.0391 | 0.0781 |

**(b) Full Core vs. NoMask, insulin dose-response slope (exploratory, uncorrected)**

| Cohort | $N$ | Mean Full Core | Mean NoMask | $W$ | $p$ |
|---|---|---|---|---|---|
| Manchester | 13 | −5.24 | −7.06 | 0.0 | 0.0002* |
| OhioT1DM | 12 | −3.37 | −7.27 | 0.0 | 0.0005* |

**Table S9:** CGM interpolation audit and interpolation-exclusion sensitivity analysis. **(a) OhioT1DM**, computed exactly from the raw XML source (before gridding), replicating the documented preprocessing rule (interior gaps $\leq$30 min/6 steps linearly interpolated; longer gaps dropped): the percentage of each participant's final (non-dropped) 5-minute grid rows that were filled by interpolation rather than an observed CGM reading. No OhioT1DM window crosses a gap longer than 30 minutes, since such rows are dropped rather than interpolated or extrapolated. **Manchester** uses a different gap-filling rule (nearest-neighbour carry-forward/backward rather than linear interpolation, with no row dropped; main manuscript, Data Preprocessing), so the Ohio percentage below is not directly transferable to Manchester; it is reported here as the quantity obtainable from the cohort whose raw pre-grid source permits an exact retrospective audit. **(b) Sensitivity re-evaluation (OhioT1DM only)**: multi-horizon rollout RMSE (mg/dL), reusing the existing corrected/original checkpoints with no retraining, computed over all rollout windows ("All") versus after excluding any window whose horizon target lands on an interpolated point ("Excl. interp."). The ordering (Persistence < Vanilla < Full Core) and the magnitude of every value are essentially unchanged, so the paper's long-horizon conclusion is robust to excluding interpolation-affected targets.

**(a) Interpolated fraction of the final CGM series, OhioT1DM (exact, from raw XML)**

| PID | 540 | 544 | 552 | 559 | 563 | 567 | 570 | 575 | 584 | 588 | 591 | 596 |
|---|---|---|---|---|---|---|---|---|---|---|---|---|
| % interpolated | 1.26 | 1.16 | 2.25 | 2.18 | 0.74 | 2.79 | 1.15 | 2.69 | 2.40 | 0.46 | 1.12 | 1.21 |

Pooled across all 165,013 retained OhioT1DM grid rows: **1.59% interpolated** (participant range 0.46–2.79%).

**(b) Rollout RMSE with vs. without interpolation-affected targets (OhioT1DM)**

| Configuration | Horizon (min) | RMSE, all windows | RMSE, excl. interp. targets | $N$, all | $N$, excl. |
|---|---|---|---|---|---|
| Persistence | 5 | 6.73 | 6.78 | 720 | 710 |
| Persistence | 30 | 24.05 | 24.14 | 720 | 710 |
| Persistence | 60 | 39.46 | 39.44 | 720 | 711 |
| Persistence | 120 | 58.42 | 58.43 | 720 | 708 |
| Vanilla | 5 | 5.91 | 5.94 | 3600 | 3550 |
| Vanilla | 30 | 22.16 | 22.22 | 3600 | 3550 |
| Vanilla | 60 | 40.23 | 40.15 | 3600 | 3555 |
| Vanilla | 120 | 67.89 | 67.99 | 3600 | 3540 |
| Full Core | 5 | 13.15 | 13.12 | 3600 | 3550 |
| Full Core | 30 | 33.74 | 33.85 | 3600 | 3550 |
| Full Core | 60 | 52.11 | 52.13 | 3600 | 3555 |
| Full Core | 120 | 72.50 | 72.83 | 3600 | 3540 |

**Table S10:** Reviewer-requested sensitivity analysis: matched no-intervention-referenced insulin dose-response ($\Delta_{rel}$) versus the pre-specified primary endpoint ($\Delta_{abs}$, unchanged). For each of the same 10 conditioning windows used for the primary insulin audit, we additionally rolled out a dose-matched no-intervention (dose = 0) trajectory from the identical starting window using the same checkpoint, and defined $\Delta_{rel}(\text{dose}) = \text{trajectory}_{\text{dose}}[-1] - \text{trajectory}_{\text{no-dose}}[-1]$, the 2-hour-ahead glucose difference between the perturbed and matched no-intervention rollout. Aggregation mirrors the primary endpoint exactly (window-averaged per dose, then slope fit by OLS). The two endpoints give the participant-averaged dose-response slope to within floating-point precision (maximum absolute difference across all cohorts, configurations, seeds, and participants: $5 \times 10^{-15}$ mg/dL per unit) for every configuration in both cohorts. This is not a coincidence: because the perturbation dose is injected into insulin-on-board only after the rollout's first prediction step, the first predicted value is dose-invariant, so $\Delta_{rel}$ and $\Delta_{abs}$ differ only by a per-window constant offset that does not depend on dose and therefore cancels exactly in the fitted slope. The matched no-intervention comparison the reviewer requested is thus mathematically guaranteed, and empirically confirmed, to yield the identical conclusion as the pre-specified $\Delta_{abs}$ endpoint.

| Cohort | Configuration | Mean slope, $\Delta_{abs}$ (primary) | Mean slope, $\Delta_{rel}$ (sensitivity) |
|---|---|---|---|
| Manchester | Full Core | −5.239 | −5.239 |
| Manchester | Vanilla | −1.978 | −1.978 |
| Manchester | NoMono | +0.074 | +0.074 |
| OhioT1DM | Full Core | −3.365 | −3.365 |
| OhioT1DM | Vanilla | −0.523 | −0.523 |
| OhioT1DM | NoMono | +1.700 | +1.700 |

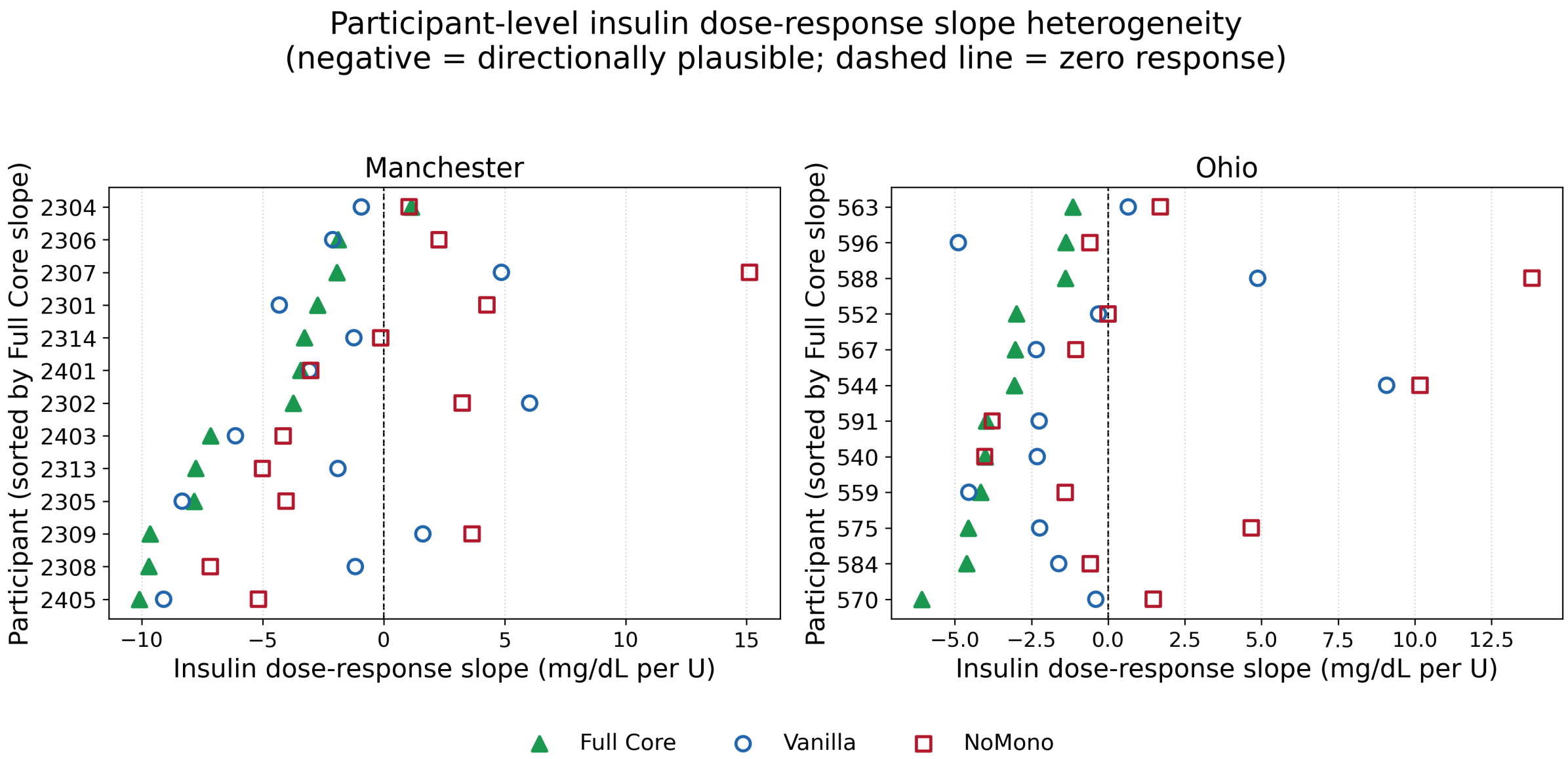


**Figure S1:** Participant-level insulin dose-response slope for every Core participant in both cohorts, for the three pre-specified primary configurations. Participants are sorted by Full Core slope within each cohort. Negative values are directionally plausible (glucose-lowering); the dashed line marks zero response. The number of participants with a wrong-signed (positive) mean slope is: Full Core 1/13 (Manchester), 0/12 (Ohio); Vanilla 3/13, 3/12; NoMono 6/13, 5/12. This figure supplements, and does not replace, the single-participant illustrative counterfactual trajectory figures in the main manuscript.
